# Tracking Single Molecule Dynamics in the Adult *Drosophila* Brain

**DOI:** 10.1523/ENEURO.0057-21.2021

**Published:** 2021-05-14

**Authors:** Adam D. Hines, Bruno van Swinderen

**Affiliations:** Queensland Brain Institute, The University of Queensland, 4072 Brisbane, Queensland, Australia

**Keywords:** brain, *Drosophila melanogaster*, *ex vivo*, general anesthesia, propofol, super-resolution microscopy

## Abstract

Super-resolution microscopy provides valuable insight for understanding the nanoscale organization within living tissue, although this method is typically restricted to cultured or dissociated cells. Here, we develop a method to track the mobility of individual proteins in *ex vivo* adult *Drosophila melanogaster* brains, focusing on a key component of the presynaptic release machinery, syntaxin1A (Sx1a). We show that individual Sx1a dynamics can be reliably tracked within neurons in the whole fly brain, and that the mobility of Sx1a molecules increases following conditional neural stimulation. We then apply this preparation to the problem of general anesthesia, to address how different anesthetics might affect single molecule dynamics in intact brain synapses. We find that propofol, etomidate, and isoflurane significantly impair Sx1a mobility, while ketamine and sevoflurane have little effect. Resolving single molecule dynamics in intact fly brains provides a novel approach to link localized molecular effects with systems-level phenomena such as general anesthesia.

## Visual Abstract

**Figure:**
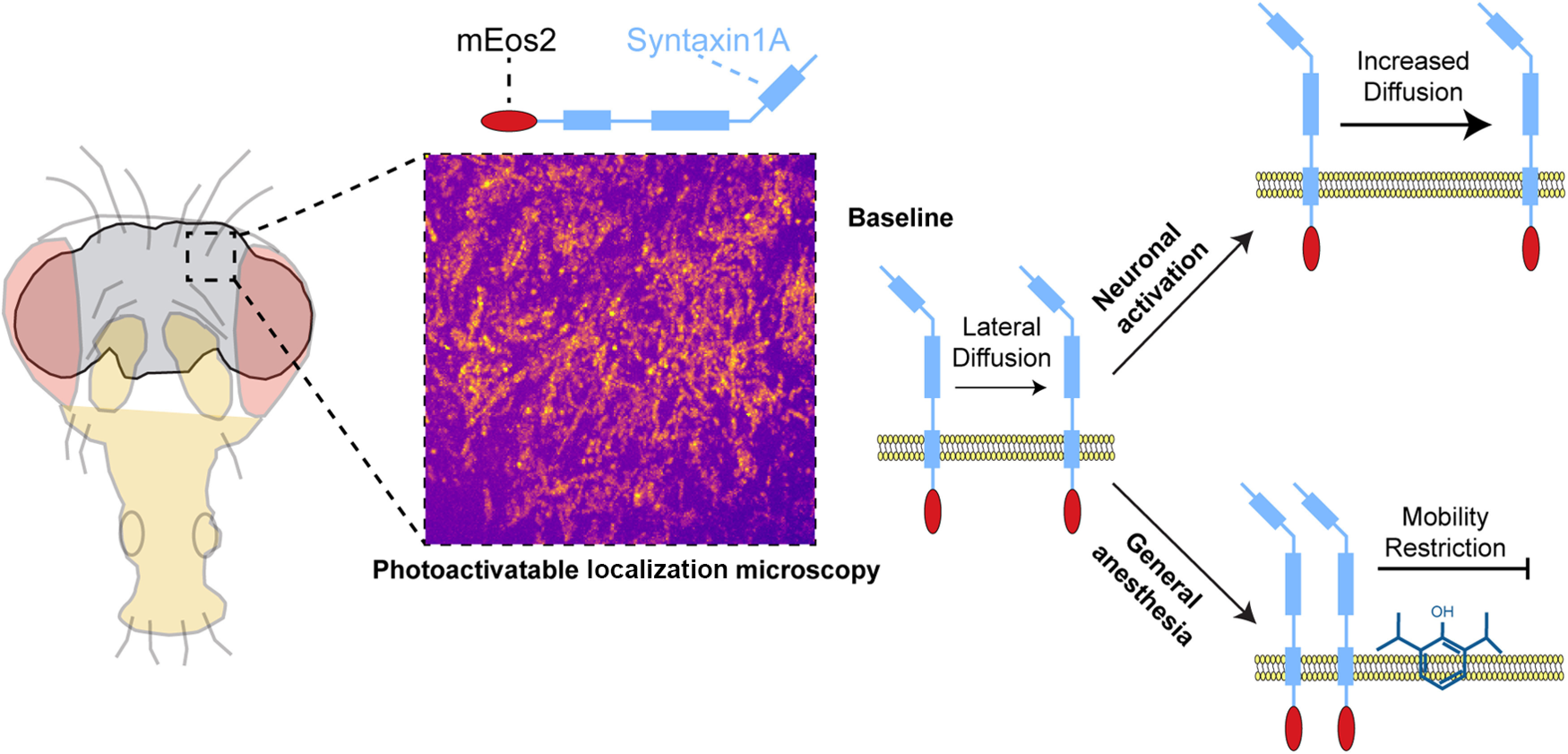

## Significance Statement

Tracking the mobility of individual syntaxin1A (Sx1a) molecules in extracted fly brains provides a physiologically-relevant context for understanding the effects of neural activation and inhibition on protein dynamics in central neurons.

## Introduction

The brain of the fruit fly *Drosophila melanogaster* offers a rich platform to explore synaptic function at multiple levels, from detailed understanding of circuits to precise molecular mechanisms of chemical neurotransmission. A key advancement aiding our understanding of neurotransmission is the development of super-resolution microscopy, which allows for the visualization of proteins and molecules below the diffraction limit of light (Betzig et al., 2006; Willig et al., 2006). Super-resolution microscopy has provided novel insight on the nanoscale structure and dynamics of key components of the presynaptic release machinery, such as syntaxin1A (Sx1a; Ullrich et al., 2015; Bademosi et al., 2016; Reddy-Alla et al., 2017). Photoactivatable localization microscopy (PALM; Betzig et al., 2006) with single particle tracking (SPT) in live cells (Manley et al., 2008) allows molecules to be detected and followed through time in a variety of systems to explore macromolecular protein dynamics (Manzo and Garcia-Parajo, 2015). This has been made possible by the development of photoconvertible fluorophores such as Eos (McKinney et al., 2009; Zhang et al., 2012), which can be attached to proteins of interest to stochastically localize molecules sparsely and thereby study protein nanoscale organization, mobility, and diffusion in cells. To study Eos-tagged proteins, dual color illumination in a total internal reflection (TIRF; Axelrod, 2001) or highly inclined and laminated optical (HILO; Tokunaga et al., 2008) sheet configuration is employed to simultaneously record and stochastically photoconvert Eos fluorophores in cultured cells or dissociated neurons (Manzo and Garcia-Parajo, 2015). However, there is comparatively little information on single molecule dynamics in more complex living tissue, such as animal brains.

Recent studies have highlighted the value of performing super-resolution microscopy and sptPALM in intact tissue, revealing, for example, developmental changes that embryos undergo by imaging single molecule dynamics in their native environment (Chen et al., 2014; Mir et al., 2018; Reisser et al., 2018; Tønnesen et al., 2018). The importance of imaging in intact, native tissue was also recently highlighted by uncovering unexpected results regarding the distribution of docked synaptic vesicles in *Drosophila* tissue compared with cultured mammalian chromaffin cells (Couteaux and Pecot-Dechavassine, 1970; Stevens et al., 2011; Jung et al., 2018). Here, synaptic vesicles in the fly larval neuromuscular junction are more readily docked and primed compared with chromaffin cells, suggesting important differences in the physiological relevance of the two systems for studying neurotransmission. We recently described single molecule imaging in intact motor nerve terminals of filleted *Drosophila* larvae (Bademosi et al., 2016, 2018a). In that study we tagged the presynaptic protein Sx1a with photoconvertible mEos2 and found that genetic stimulation of motoneurons resulted in increased mobility of Sx1A in the motor nerve terminals, suggesting increased mobilization of the presynaptic machinery when neurons are activated. In contrast, stimulation of chromaffin cells results in decreased Sx1a mobility (Kasula et al., 2016), highlighting that even highly conserved molecular mechanisms can differ depending on tissue type.

Sx1a is necessary for the docking and fusion of neurotransmitter-containing vesicles, and is a component of the SNARE complex along with its binding partners SNAP25 and VAMP2 (Südhof, 2012). Sx1a function is highly conserved in all animals (Bennett et al., 1992; Ferro-Novick and Jahn, 1994; Südhof and Rizo, 2011), with mutations in the protein often implicated in synaptic communication defects and lethality (Schulze et al., 1995; Saifee et al., 1998; Fergestad et al., 2001; Fujiwara et al., 2006; Vardar et al., 2016; Kofuji et al., 2017). Our growing understanding of the mechanisms underlying synaptic function has uncovered novel hypotheses for how neurotransmission might be compromised by certain drugs, such as general anesthetics (Hemmings et al., 2005, 2019; Humphrey et al., 2007; van Swinderen and Kottler, 2014; Baumgart et al., 2015; Bademosi et al., 2018b; Troup et al., 2019; Karunanithi et al., 2020). A Sx1a gain-of-function mutation was found to confer resistance to volatile general anesthetics in the nematode *Caenorhabditis elegans* (van Swinderen et al., 1999) as well as *Drosophila* flies (Troup et al., 2019), suggesting a potential presynaptic target mechanism for these drugs. Single molecule imaging of mEos-tagged Sx1a in *Drosophila* motor nerve terminals exposed to the sedative drug propofol revealed that this common general anesthetic may be immobilizing Sx1a into nanoclusters (Bademosi et al., 2018b). Thus, motor neuron activation and propofol exposure appeared to have opposite effects on Sx1a mobility in intact synapses, although these experiments were restricted to relatively large motor nerve terminals, so the relevance to smaller synapses in the brain remains unknown.

Here, we adapt super-resolution imaging and SPT techniques to the extracted adult fly brain and use this approach to determine whether Sx1a mobility can be acutely modulated in central synapses. Along with employing a thermogenetic neural activation paradigm, we test a panel of intravenous and volatile general anesthetics for potential effects on Sx1a mobility. We find that, similar to *Drosophila* larval neuromuscular junction (Bademosi et al., 2016), the mobility of Sx1a molecules in the adult brain is increased on neuronal stimulation, thereby providing a physiologically relevant setting to probe for general anesthetic effects in intact brain tissue.

## Materials and Methods

### Fly stocks and rearing conditions

Sx1a-mEos2 transgenic fly lines were generated as previously described (Bademosi et al., 2018a). Briefly, Sx1a cDNA was cloned to include a mEos2 tag by replacing the stop codon of Sx1a with a linker molecule GAGGTACCGCGGGCCCGGGATCCACCG. Whether mEos2 is appropriate for a C-terminal or N-terminal attachment depends on the protein of interest to study. Sx1a-mEos2 flies were injected with phiC31 onto the second chromosome and balanced with curly (Cyo). For dTrpA1 (Drosophila transient receptor potential cation channel 1a) experiments, w^1118^;Sx1a-mEos2/Cyo;+/+ flies were crossed to a w^1118^;+/+;UAS-dTrpA1 line to generate a stable breeding stock with the genotype w^1118^;Sx1a-mEos2/Cyo;UAS-dTrpA1.

*D. melanogaster* fruit flies were reared on standard yeast-sugar-agar food in vials at 22°C with a 12/12 h light/dark cycle. w^1118^;Sx1a-mEos2/Cyo;UAS-dTrpA1 transgenic lines were crossed with w^1118^;+/+;R57C10-Gal4 virgin females to generate the w^1118^;Sx1a-mEos2/+;UAS-dTrpA1/R57C10-Gal4 flies which were used throughout this study. Flies were raised at 19°C after which point females of the required genotype were collected under brief CO_2_ exposure and then kept at 19°C on a 12/12 h light/dark cycle for 3–5 d before experiments. Keeping the flies at 19°C prevented activation of dTrpA1 channels. The effectiveness of dTrpA1 was confirmed by exposing flies briefly to 30°C, which rapidly induced paralysis (Movie 4).

### Imaging solution

Modified hemolymph-like 3.1 (HL3.1) solution was prepared fresh on the day of an experiment and used both as a dissecting and imaging buffer. HL3.1 consists of 70 mm NaCl, 5 mmKCl, 1.5 mmCaCl_2_, 2 mm MgCl_2_, 5 mm HEPES, 115 mm sucrose, 5 mm trehalose, and pH 7.2 with NaHCO_3_ (Sigma-Aldrich).

Modified hemolymph-like 3 (HL3) solution used in Extended Data Figure 3-1 consisted of 70 mm NaCl, 5 mmKCl, 1.5 mm CaCl_2_, 20 mm MgCl_2_, 5 mm HEPES, 115 mm sucrose, 5 mm trehalose, and pH 7.2 with NaHCO_3_ (Sigma-Aldrich). Artificial CSF (aCSF) contained 25 mm HEPES, 120 mm NaCl, 5 mmKCl, 2 mm CaCl_2_, 2 mm MgCl_2_, and 30 mm glucose buffered to a pH of 7.4 using NaOH.

### Anesthetics

All anesthetic drugs were diluted into HL3.1 and mixed by vigorous vortexing for ∼1 min. For intravenous anesthetics, except for ketamine, these were first diluted from stock in dimethyl sulfoxide (DMSO, Sigma-Aldrich D5879-500Ml). Relevant concentrations were determined as previously described but not matched for equipotency (Zalucki et al., 2015; Bademosi et al., 2018b). Volatile anesthetics were taken directly from a stock bottle using a 10 μL Hamilton syringe (Hamilton Company). A fresh preparation of HL3.1 solution with volatile anesthetics was made for each dissection. Estimates of isoflurane and sevoflurane concentrations were based on previous work (Sandstrom, 2004; Zalucki et al., 2015): 3 and 6 μl of 100% stock of isoflurane and sevoflurane were each diluted into 20 mL of HL3.1 solution, which correspond to ∼0.19 and 0.38 mm, respectively, based on chromatography results from multiple HL3.1 samples (Zalucki et al., 2015; Bademosi et al., 2018b). The following anesthetics were used: 2,6-diisopropylphenol (propofol; Sigma-Aldrich D126608-100G), etomidate (Sigma-Aldrich, E6530-10MG), ketamine (Ilium Ketamil, Provet), isoflurane (Henry Schein, 1182097), and sevoflurane (Fluorochem, 28523-86-6)

### Dissection of *Drosophila* brains

The brains of 3- to 5-d old female *Drosophila* flies were removed using a standard dissecting technique (Wu and Luo, 2006) on a Sylgard (Dow Corning) dish after brief anesthesia on a CO_2_ pad. Females were chosen to keep sexual dimorphisms consistent between experiments. Using Dumont #5 forceps (Fine Science Tools, 11251-10), heads were removed from the body and placed in HL3.1 solution. The proboscis was then removed to gain access to the inside of the cuticle. Carefully tearing away at the cuticle until the brain is released, the brains were cleared of all tracheal tissue. Dissected brains were then mounted in ∼10 μl of HL3.1 on a glass slide (Superfrost, ThermoFisher), and sealed shut using a 25-mm square cover glass (Menzel–Gläser, ThermoFisher) rimmed with silicone vacuum grease (Dow Corning) with a paintbrush. For fixed brain imaging, brains were dissected as usual and then fixed in 4% paraformaldehyde (PFA) for 40 min and then washed in HL3.1 solution. Brains were then mounted in the same manner and imaged.

### Super-resolution and PALM

All imaging was performed on a standard Zeiss ELYRA PS.1 microscope fitted with a Zeiss Plan-APOCHROMAT 100 × 1.4 nA oil immersion objective, a Zeiss FC12 definite focus, and an iXon EMCCD 512 × 512-pixel camera (Andor, Oxford Instruments). Mounted brains were inverted so that the oil-objective touches the coverslip and the region of interest (ROI) was navigated visually using bright-field illumination. Brains were imaged at a HILO sheet angle of 47.3° to improve the signal-to-noise ratio, with a 1.6× lens magnification, in TIRF high power mode. A 570–620 + 750 filter cube was employed to further improve the signal. In order to simultaneously photoconvert native mEos2 and record photoconverted particles, two lasers with 405 and 561 nm wavelengths, respectively, were used to perform PALM. The laser powers used were 25% of the 561-nm laser, with an average power at the specimen of 0.21 mW; 405-nm laser power varied with different recordings, from 0.001% to 0.01% with a power at the specimen of 0.1 μW. Because of a high amount of auto-photoconversion that occurs in the bright-field light from brain dissections, we first allowed the photoconverted particles to bleach for ∼1 min without the 405-nm illumination to establish a baseline. Drift during imaging was evaluated per brain at this step by finding stable bright spots, which are likely auto-fluorescing protein aggregates of unknown providence. With the 561-nm illumination, an ROI was drawn around the spot, followed by 3 min of continuous recording to see whether the spot moved out of the ROI. Drift was also evaluated after imaging using a Pearson cross-correlation of the maximum z-projection of the 25°C and 30°C recordings (Extended Data Fig. 3-3). Details of the Pearson calculations are described in the Data and Statistical Analysis section. Brains that drifted were discarded. Drift can often occur because of the movement of recording solution toward the periphery of the coverslip, which can be overcome by sealing the coverslip edges with silicone grease, decreasing the size of the coverslip or increasing the amount of imaging solution. Zeiss Zen 2012 software was used to set the imaging parameters and capture the recordings.

For dTRPA1 activation experiments, a Zeiss incubation chamber, Heating Unit XL S, and TempModule S (Zeiss) was used to set, change, and monitor recording temperatures. An initial baseline recording at 25°C was taken for all experiments (unless noted otherwise) which was then increased to 30°C to stimulate neurotransmission and perform a second recording at the same location. The power of the ultraviolet (UV)-405-nm laser was adjusted throughout recordings to maintain the number of stochastically switched mEos2 molecules. A minimum of 16,000 frames were captured at 30-ms frame rate with continuous exposure, the lowest exposure time achievable with the hardware used, to ensure at least 1000 Sx1a-mEos2 trajectories were recorded per experiment.

### Western blotting

20 × w^1118^;Sx1a-mEos2/+;UAS-TrpA1/R57C10-Gal4 female flies aged 3- to 5-d old were briefly anesthetized on a CO_2_ pad and sorted before transferring to a 15-ml falcon tube on dry ice. Flies were vortexed for 15 s twice to separate the heads from the body. No. 25 and No. 40 standard sieves (Endecotts Ltd.) prechilled at −80°C were used to separate heads from the body and legs. Heads were collected into a prechilled 1.5 ml Eppendorf tube on dry ice with 30 μl of a 1% Triton X-100 lysis buffer containing a 1:100 EDTA-free protease inhibitor cocktail. Heads were homogenized with a 1/4” ceramic sphere (MP, catalog #6540–034) in a QIAGENTissueLyser LT. Homogenate was centrifuged for 20 min at 14,000 rpm at 4°C to separate cellular debris from the lysate. The lysate was then added to 2× SDS loading buffer and boiled for 10 min at 100°C; 30 μl of the boiled sample was immediately loaded into a Mini-PROTEAN TGX 4–15% gel (Bio-Rad catalog #456-1083) and separated at 110 V. Gel was then transferred onto an Immobilon-P membrane (Merck, catalog #IPVH00010) at 100 V. The membrane was blocked in TBST (TBS + 1% Tween) solution containing 5% milk for 1 h at room temperature and washed 3× with TBS after which it was incubated overnight at 4°C with an anti-Sx1a antibody (Developmental Studies Hybridoma Bank, catalog #8C3) diluted 1:1000 in a TBST solution with gentle agitation. The following morning, the membrane was washed 3× in a TBST solution containing 1% milk and incubated with a secondary antibody (goat to mouse IgG HRP, Abcam catalog #ab205719) in a 1:10,000 dilution for 1 h. Membranes were washed 3× in TBS and visualized in a Pierce ECL Western blotting substrate (Thermo-Scientific, catalog #32106) using a Li-cor Odyssey Fc. Protein was quantified using Image Studio Lite (LI-COR Biosciences).

### Data and statistical analysis

All data were analyzed using the free Fiji software TrackMate (Tinevez et al., 2017) adapted into a custom MATLAB GUI called single particle analysis (SPA; available from https://github.com/AdamDHines/sptPALM-Analysis) which incorporates mean squared displacement (MSD) and diffusion coefficient calculations, performed on a Lenovo ThinkPad with Windows 10. The analysis guide is available as Extended Data Document 1. Single Sx1a-mEos2 molecules were localized using a Laplacian of Gaussian (LoG) detection algorithm, median filtering, and subpixel localization with a manually determined threshold value for each recording:

(1) $$g\left(x,y,t\right)=\frac{1}{2\pi t}e^{-\frac{x^{2}+y^{2}}{2t}}.$$

To track single molecules between frames, a linear assignment problem (LAP) algorithm (Jaqaman et al., 2008) was used to link particles by minimizing a cost matrix of distance between detected particles in a frame to every particle in the next frame. A minimum of 6 and a maximum of 1000 spots per track were included for analysis of the MSD, which measures the distance a particle travels from its initial position and is calculated by:

(2) $$MSD\left(n\times \Delta t\right)=\overset{N-n}{\underset{i=1}{\sum }}\frac{{\left[x\left(\left(i+n\right)\times \Delta t\right)-x\left(i\times \Delta t\right)\right]}^{2}+{\left[y\left(\left(i+n\right)\times \Delta t\right)-y\left(i\times \Delta t\right)\right]}^{2}}{N-n}.$$

10.1523/ENEURO.0057-21.2021.ed1 Extended Data Document 1 sptPALM analysis guide. Download Extended Data Document 1, DOCX file.

The diffusion coefficient, *D*, was calculated for each MSD curve with linear fits of the first four time points using the following:

(3) MSD(τ)=α + 4Dτ.

*N* is the number of data points, the offset constant α includes the effects of localization error and finite camera exposure, Δ*t* is the time interval between each frame, with *x* and *y* being spatial coordinates for localizations in each image. Mobile-to-immobile ratios were calculated by summing the relative frequency of molecules with a log_10_ diffusion coefficient of more and less than −1.6 and dividing the mobile by the immobile fraction, which translates to be 0.021 μm^2^ s^−1^ (Constals et al., 2015).

The point spread function (PSF) half width and localization precision of Sx1a-mEos2 molecules was determined from a single brain that was fixed in 4% PFA for 45 min before imaging using Zeiss ZEN 2012 software (Extended Data Fig. 1-4).

For all experiments using thermogenetic stimulation, the peak MSD value for the baseline condition was used to normalize all values of the MSD (Watts et al., 2014) for both unstimulated and stimulated conditions, such that the peak MSD value for the unstimulated condition was set to 1 (Extended Data Fig. 3-2). Diffusion coefficients and mobile-to-immobile ratios were not able to be calculated with normalized MSD curves. Pearson correlations were used to determine levels of drift by comparing the maximum z-projection of the 25°C and 30°C recordings to calculate Pearson coefficients, performed in ImageJ using the colocalization threshold function (Extended Data Fig. 3-3). The peak mobility point (0.30 s) of the normalized 30°C data were subtracted by the peak point of the 25°C internal control to derive δ mobility (Extended Data Fig. 3-3). The δ mobilities were plotted against calculated Pearson coefficients to develop a linear regression and derive an *R*^2^ value. The area under the curve (AUC) was measured for each normalized MSD curve using GraphPad Prism 8, with a baseline starting at Y = 0, ignoring peaks that are <10% of the distance from minimum to maximum Y, and defining that all peaks must go above baseline. To compare the mean of internally controlled AUC values a Wilcoxon matched signed-rank test was used with a significance threshold of *p* = 0.05. To compare the means of the AUC of different conditions to controls, a Kolmogorov–Smirnov test with a significance threshold of *p* = 0.05 was used. MSD presented is ±SD and AUC data are ±5–95th percentile. 95% confidence intervals (CIs) were calculated around the mean.

### Data availability

The datasets supporting the current study will be made available on a public database (eSpace, The University of Queensland) on publication: https://espace.library.uq.edu.au/.

### Code accessibility

The code/software described in the paper is freely available online at https://github.com/AdamDHines/sptPALM-Analysis.

## Results and Discussion

### Localizing and tracking the mobility of Sx1a in the adult fly brain

**Figure 1.**
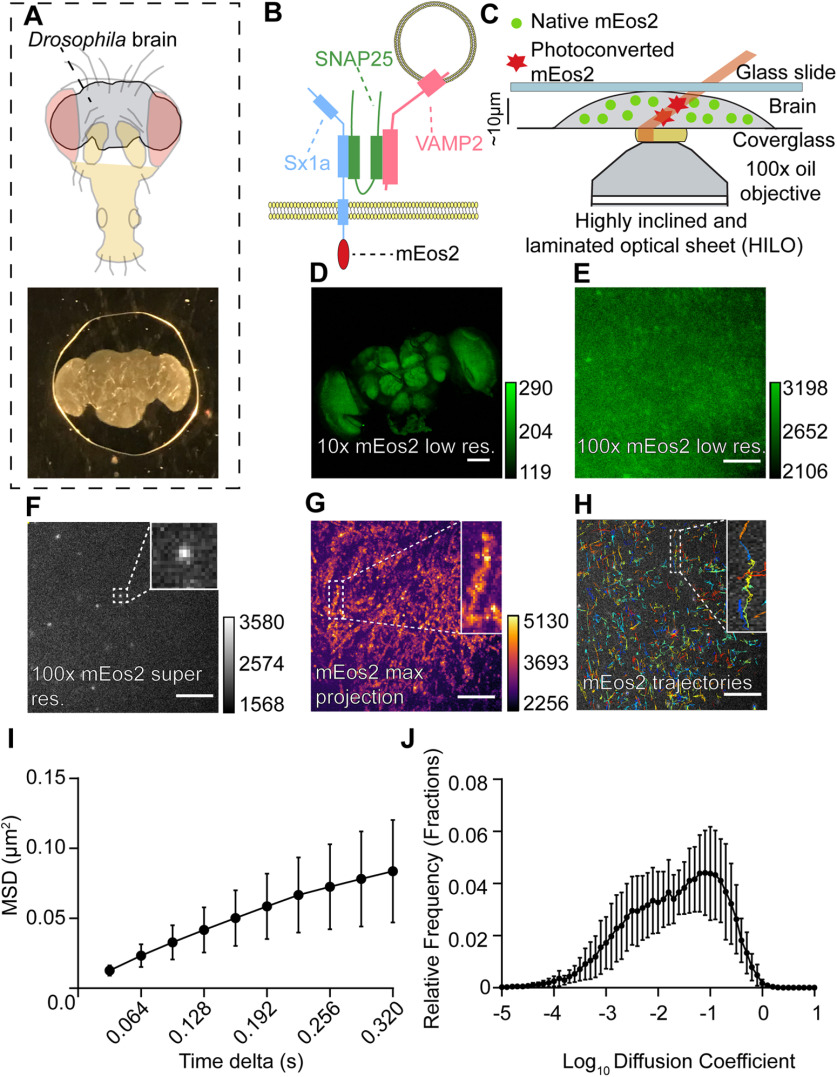
Imaging single Sx1a molecules in adult *Drosophila* brains. ***A***, The brain of adult *Drosophila* fruit flies (top) is dissected and mounted in a HL3.1 buffer and sealed between a glass slide and coverslip (bottom). ***B***, Schema of the protein of interest being imaged, Sx1a (blue), with its SNARE partners SNAP25 (green), and VAMP2 (red). Sx1a is tagged with the photoconvertible fluorophore mEos2 on the C terminus. ***C***, Sx1a-mEos2-expressing brains are imaged under a HILO sheet illumination with simultaneous UV-405-nm photoconverting and 561-nm recording lasers. ***D***, A 10× confocal image showing expression of mEos2 (native non-photoconverted green form) across the entire fly brain (scale bar: 100 μm, right calibration scale). ***E***, Green form of mEos2 expression at 100× magnification, individual molecules cannot be resolved because of PSF overlap (scale bar: 5 μm, right calibration scale). ***F***,Stochastically photoconverting mEos2 with a UV-405-nm laser can resolve single Sx1a-mEos2 molecules using a 561-nm laser without any PSF overlap (scale bar: 5 μm, right calibration scale). Inset, digital zoom of one molecule. ***G***, Neuropil ultrastructure in the fly brain becomes apparent following a maximum intensity projection of all photoconverted mEos2 molecules over 16,000 frames of acquisition (scale bar: 5 μm, right calibration scale). Inset, digital zoom of one neuronal compartment. ***H***, SPT is performed on all detected Sx1a-mEos2 to track individual Sx1a molecules. Inset, Individual trajectories in different colors. ***I***,***J***, Analysis of Sx1a-mEos2 trajectories reveals the mobility of Sx1a-mEos2 by calculating the MSD and diffusion coefficients of single trajectories (*n *= 13 brains, data are ± SD). See Extended Data Figures 1-1, 1-2, 1-3, 1-4, 1-5.

We employed sptPALM to image and track individual Sx1a molecules in the *ex vivo* brains of adult *Drosophila* fruit flies (Fig. 1*A*; Extended Data Fig. 1-1). *Ex vivo* fly brains in buffer solutions remain viable and physiologically healthy for several hours (Gu and O’Dowd, 2006; Raccuglia et al., 2019), allowing us to apply this preparation to live-cell microscopy. Sx1a was tagged on the extracellular C terminus with the photoconvertible fluorophore mEos2 (McKinney et al., 2009) and expressed pan-neuronally (Bademosi et al., 2016). Importantly, Sx1a-mEos2 expression was low relative to endogenous Sx1a in the adult fly brain (Extended Data Fig. 1-2), consistent with previous findings in larvae (Bademosi et al., 2016). Brains were mounted onto a glass slide in ∼10 μl of fresh modified hemolymph-like solution 3.1 (HL3.1; Feng et al., 2004) and sealed with a square coverslip (Menzel–Gläser, ThermoFisher) rimmed with vacuum grease (Dow Corning; Fig. 1*A*, lower). Light compression reduced the thickness of the brain from ∼120 to 40 μm, allowing for the imaging of tissue in a HILO configuration while retaining neural circuit architecture (Fig. 1*C*; Extended Data Fig. 1-3). Spinning disk confocal imaging confirmed mEos2 expression in brain neurons (Fig. 1*D*). When observing the brain at 100× magnification, the PSF overlap of the unconverted green form of mEos2 does not allow for the resolution of individual molecules or structures within the fly brain (Fig. 1*E*). Upon exposure to a low intensity UV (405 nm) photoconverting stimulus, stochastically switched red mEos2 molecules can be visualized sparsely (Fig. 1*F*). In order to confirm that we were imaging mEos2 molecules, we compared spot counts in brains that had no UV exposure and saw a significant increase in single molecule detection with photoconversion (Extended Data Fig. 1-4). At 30-ms exposure time, Sx1a-mEos2 molecules can be seen moving inside of neurons of the fly brain (Movie 1). We were able to achieve a localization precision of ∼18 nm, which is close to previously reported measures (Extended Data Fig. 1-4; McKinney et al., 2009). Neural structures in the fly brain become evident after performing a maximum projection of a time series of PALM experiments (Fig. 1*G*), confirming that Sx1a-mEos2 is confined.

10.1523/ENEURO.0057-21.2021.f1-1 Extended Data Figure 1-1 SPT and PALM. ***A***, ***B***, Raw image sequences from PALM (***A***) are processed with a LoG convolution filter (***B***) for automatic spot detection to derive centroids of single Sx1a-mEos2 particles. ***C***, ***D***, Schematic of tracking particles in a 2D sample over time, with links between frames determined based on the relative distance (δ, distance) of a single particle to every other particles (***D***) from one frame to the next. ***E***, Particle tracking is solved using a LAP cost matrix, where the cost is the relative distance of a particle in frame *n* to every other particle in frame *n* + 1. A particle in frame *n* can have one of four outcomes based on the localization in the proceeding frame. A particle has a potential link (λ) to another particle based on a maximum linking distance which if a particle in the proceeding frame exceeds becomes an impossible link (x). To avoid linking potentially unrelated molecules, it is important to keep stochastic switching of fluorophores light such that molecules detection is sparse. The threshold for the maximum linking distance depends on a variety of factors, including the exposure time of the imaging and the relative speed of the molecule, and if it is membrane bound or cytoplasmic. A particle can also either be the start or the end of a trajectory, and a higher cost value is employed to determine whether a particle should be linked to another particle or not (α and β). ***F***,Example of the cost matrix used to solve SPT. The matrix is solved for least cost to link particles and determine whether a trajectory is at its beginning or its end (adapted from Jaqaman et al., 2008). Download Figure 1-1, TIF file.

**Figure 2.**
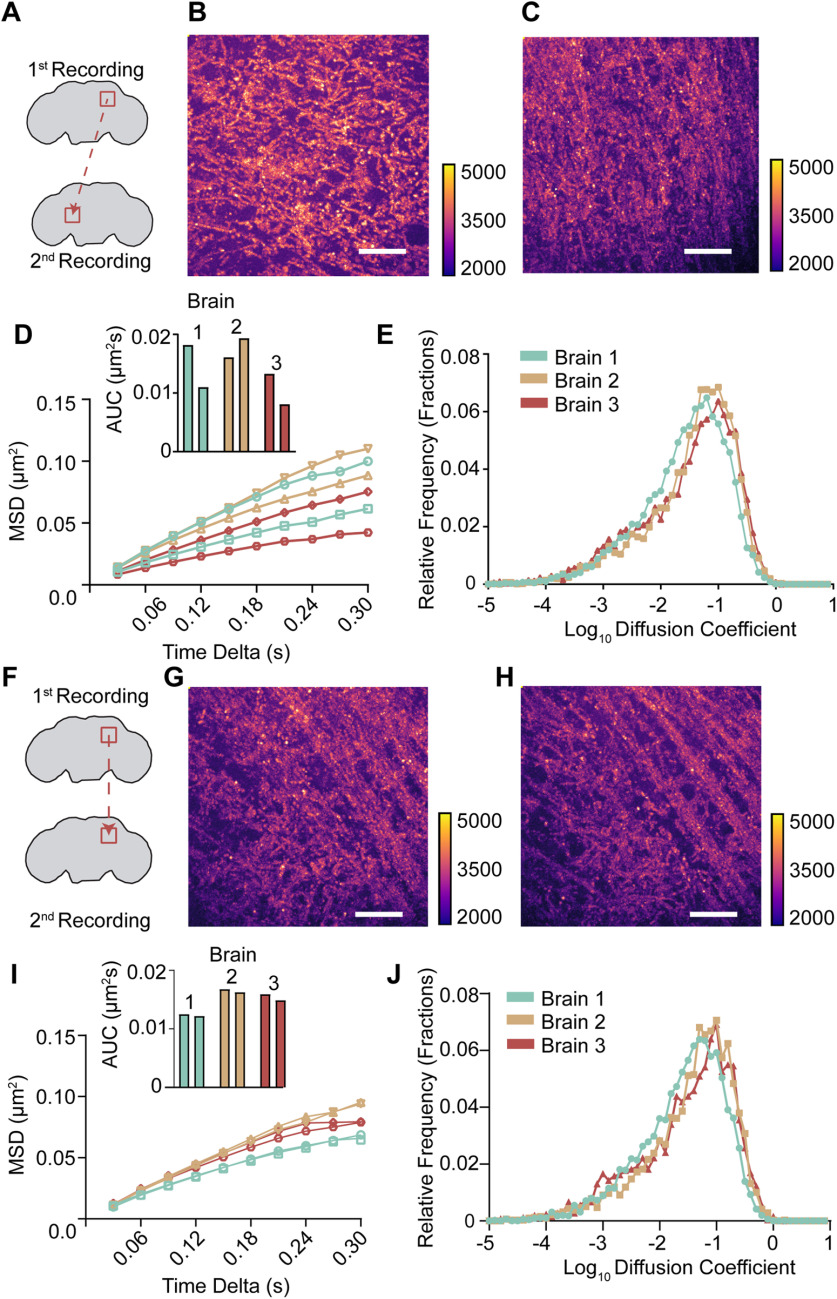
Tracking Sx1a-mEos2 mobility from the same brain region is highly reproducible. ***A***, Two recordings were taken from different regions in the same brain to establish whether differences in Sx1a-mEos2 mobility are observed. ***B***,***C***, Maximum stack projections as in Figure 1*G* for the two distinct brain regions as shown in ***A*** reveals different distribution of Sx1a-mEos2 molecules. ***D***, The MSD and AUC for two successive recordings in three separate brains highlights that within a brain there are different levels of Sx1a-mEos2 mobility, resulting in different diffusion coefficient estimates (***E***) across experiments. ***F***,Two recordings were taken from the same brain region, to determine whether Sx1a-mEos2 mobility was consistent. ***G***,***H***, Maximum stack projections for the same brain region recorded twice highlights that the neuronal structure remains the same. ***I***, When Sx1a-mEos2 is tracked in the same region twice, the MSD and AUC remain consistent, with similar diffusion coefficients (***J***), providing a framework for internally controlled experiments performed at the same recording site. Scale bars: 5 μm, calibration scales to the right of each. See Extended Data Figure 2-1.

10.1523/ENEURO.0057-21.2021.f1-2 Extended Data Figure 1-2 Quantification of Sx1a-mEos2 expression relative to endogenous Sx1a. ***A***, 20× female heads from experimental flies (w^1118^;Sx1a-mEos2/+;UAS-TrpA1/R57C10-Gal4) aged 3–5 d were homogenized and run in a Western blot on an SDS-PAGE gel (***B***) to separate endogenous Sx1a protein (arrow at 36 kDa) from Sx1a-mEos2 (arrow at 60 kDa). ***C***, Quantification of the relative expression of Sx1a-mEos2 compared to endogenous Sx1a shows approximately 8% of the expression level of Sx1a-mEos2. Download Figure 1-2, TIF file.

**Figure 3.**
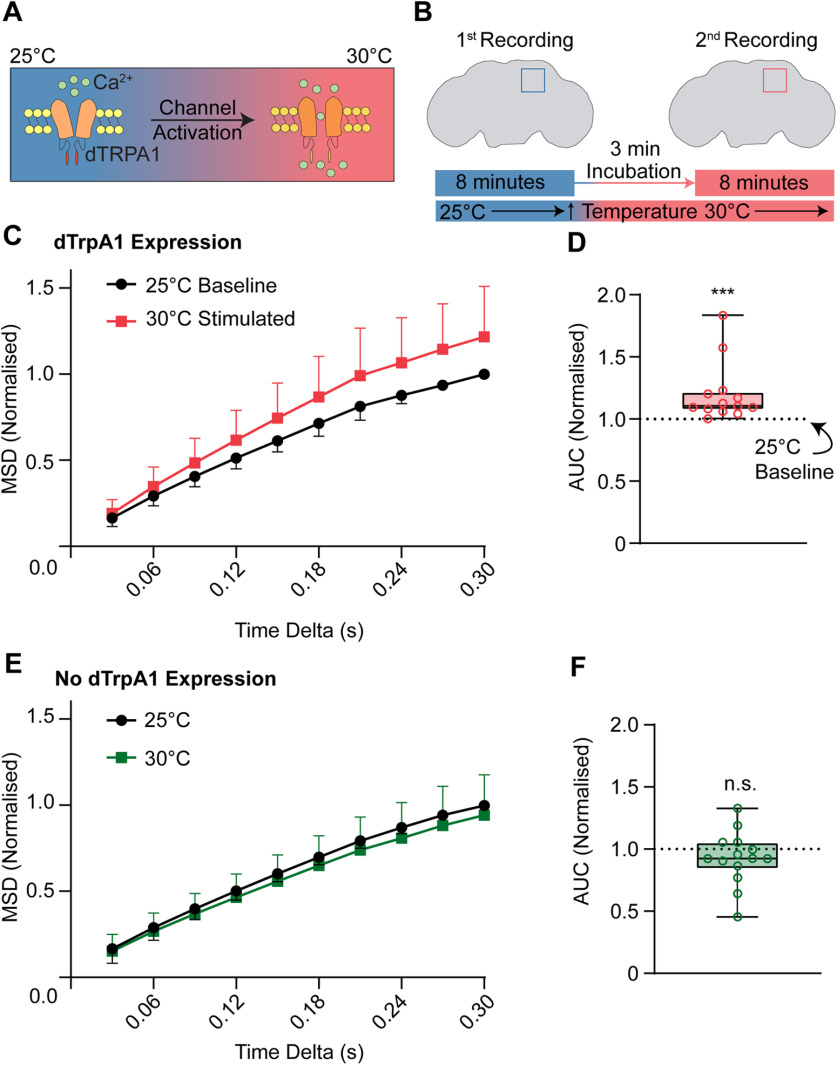
Neuronal stimulation increases the mobility of Sx1a-mEos2. ***A***, Schematic of the *Drosophila* transient receptor potential cation channel type A1 (dTrpA1) function. At 25°C dTrpA1channels remain closed; increasing ambient temperature to 30°C activates these channels, resulting in Ca^2+^ influx and neuronal depolarization. ***B***, To measure the effects of dTrpA1 activation in the fly brain, recordings were taken from the same brain region twice: baseline recording was at 25°C followed by recording at 30°C after increasing incubation temperatures. ***C***,***D***, dTrpA1 stimulation of fly neurons increased the mobility of Sx1a-mEos2 molecules compared with baseline (dotted line). All experiments were normalized to their own internal control at 25°C (*n *= 13, *p* = 0.0002, Wilcoxon test; AUC, AUC 95% CI 1.059–1.347, data for MSD is ± SD, data for AUC is ±5–95th percentile). ***E***,***F***, In the absence of the R57C10-Gal4 driver, no dTrpA1 was expressed in fly neurons and Sx1a-mEos2 mobility was not increased at 30°C (*n *= 14, *p* = 0.1531, AUC 95% CI 30°C 0.8029–1.051, Wilcoxon test, data for MSD is ± SD, data for AUC is ±5–95th percentile). See Extended Data Figures 3-1, 3-2, 3-30. n.s., not significant. ***p < 0.001.

10.1523/ENEURO.0057-21.2021.f1-3 Extended Data Figure 1-3 Internal brain structures remain intact and are better resolved in a compressed preparation. ***A***, When the fly brain is not compressed (thickness = 120 μm), light scattering under a HILO sheet setting decreases the resolution of imaged structures. ***B***, When the brain is lightly compressed (thickness = 40 μm), the scattering interferes less, and structures are more resolved. ***C***, Fly brain expression of Sx1a-mEos2 in an un-compressed preparation shows a distinct lack of neuronal architecture compared to a compressed preparation (***D***). Red box in ***D*** indicates where internally controlled imaging experiments were conducted, in the general vicinity of the lateral protocerebrum.***E***, ***G***, 10× and 63× oil magnification, respectively, of UAS-CD8GFP>R23E10-Gal4 (Jenett et al., 2012) imaging in a standard uncompressed preparation. ***F***, ***H***, 10× and 63× oil magnification, respectively, of UAS-CD8GFP>R23E10-Gal4 imaging in a compressed preparation, revealing that neural architecture of a defined circuit in the fly brain (the dorsal fan-shaped body) remains intact. Download Figure 1-3, TIF file.

**Figure 4.**
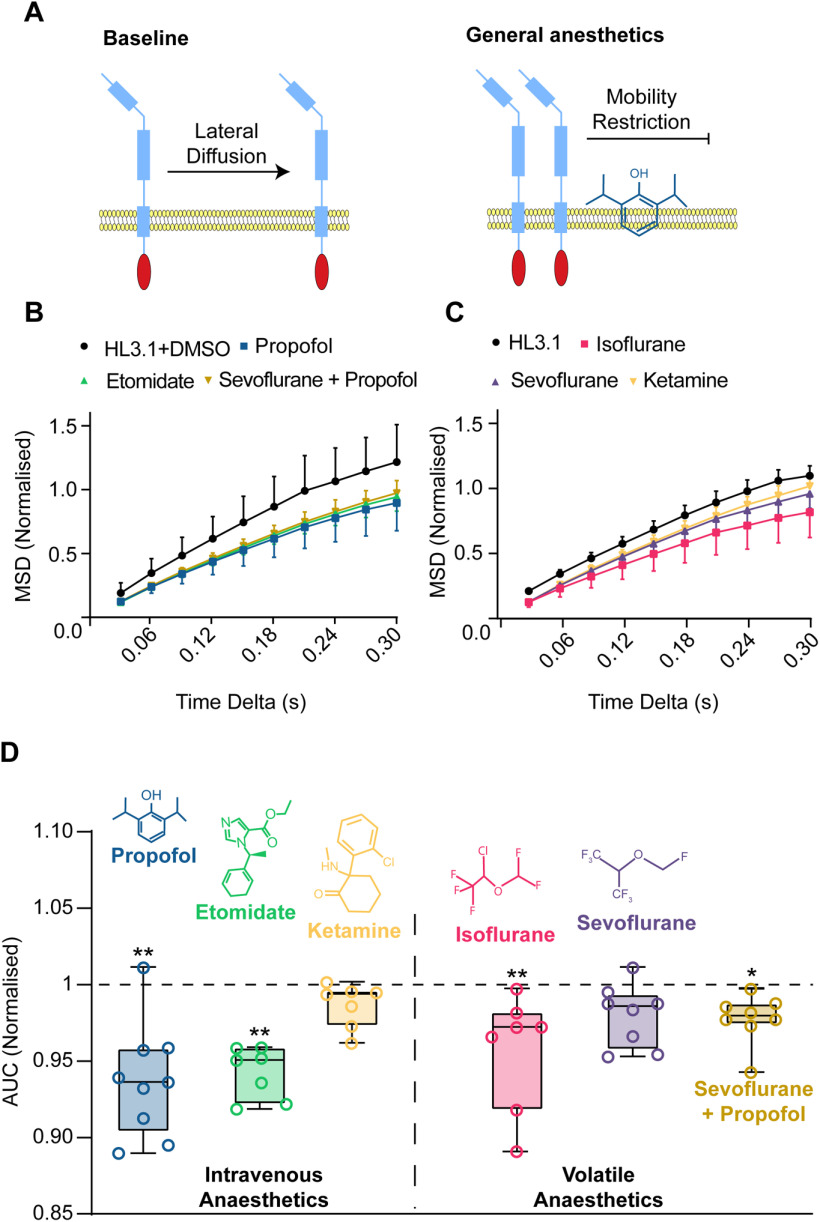
General anesthetics restrict Sx1a-mEos2 mobility in adult *Drosophila* brains. ***A***, left,Sx1a-mEos2 is able to diffuse laterally across a membrane, but mobility becomes restricted in the presence of propofol (right). ***B***, Normalized MSD curves comparing all anesthetics under stimulation that contained DMSO in the HL3.1. ***C***, Same as ***B*** but without DMSO in the solution (MSD is normalized). All data are represented as ±SD. ***D***, Intravenous and volatile general anesthetics restrict the mobility of Sx1a-mEos2 compared with respective controls (dashed line). Both propofol (3 μm) and etomidate (8 μm) significantly reduced Sx1A-mEos2 mobility (AUC) when compared with a HL3.1+DMSO control (propofol *n *= 9, *p* = 0.0009, AUC 95% CI 0.908–0.966; etomidate *n *= 8, *p* = 0.0055, AUC 95% CI 0.927–0.958, Kruskal–Wallis test, data are ±5–95th percentile). Ketamine (100 μm) was unable to restrict Sx1a-mEos2 mobility when compared with a HL3.1 control (*n *= 6, *p* = 0.9924, AUC 95% CI 0.974–1.00, Kruskal–Wallis test, data are ±5–95th percentile). The volatile anesthetic isoflurane (0.19 mm) was able to restrict Sx1a-mEos2 mobility but sevoflurane (0.38 mm) was not, compared with a HL3.1 control (isoflurane *n *= 9, *p* = 0.0079, AUC 95% CI 0.922–0.992; sevoflurane *n *= 8, *p* = 0.2672, AUC 95% CI 0.963–0.998, Kruskal–Wallis test, data are ±5–95th percentile). The addition of propofol (3 μm) to sevoflurane significantly restricted Sx1a-mEos2 mobility compared with a HL3.1+DMSO control (*n *= 8, *p* = 0.0108, AUC 95% CI 0.965–0.992). See Extended Data Figures 4-1, 4-2. *p < 0.05; **p < 0.01.

10.1523/ENEURO.0057-21.2021.f1-4 Extended Data Figure 1-4 Analysis of Sx1a-mEos2 localizations in the adult *Drosophila* brain. ***A***, Single frame from a recording in the fly brain with no 405-nm photoconverting laser while imaging red 561 nm and (***B***) with the 405-nm photoconversion reveals a (***C***) significant increase in spot detection (*n *= 6, average spot detection 561 nm: 54,787 spots, 95% CI 40,901–68,672; average spot detection 405 nm + 561 nm: 74,663 spots, 95% CI 57,049–92,276, *p *=* *0.0313, Wilcoxon test, data is ±5–95th percentile). Spot counts were recorded in the same brain in the same region twice over the course of 8000 frames without the 405-nm laser and another 8000 frames with the 405-nm laser. To analyze the characteristics of Sx1a-mEos2 localizations, recordings in HL3.1 were utilized. Using Zeiss processing software ZEN, we processed the acquired images with PALM, which measured the (***D***) PSF half width (black line is the average, SD shown in gray) and (***E***) the localization precision of detected Sx1a-mEos2 molecules. On average, the PSF half width was 127.9 ± a SD of 4.725 nm (*n *= 10, 95% CI 124.5–131.3 nm), and we achieved on average a localization precision of 18.1 ± a SD of 2.5 nm (*n *= 10, 95% CI 16.3–19.90 nm). In order to quantify the average number of localizations per frame and trajectory length for Sx1a-mEos2 molecules, we utilized our tracking software SPA (see Materials and Methods). On average, we detected 9.8 ± 2.6 (SD) molecules per frame (*n *= 10, 95% CI 7.9–11.7) with the majority of trajectory lengths from detected molecules being eight frames long, the minimum required for analysis. All box plots are ±5–95th percentile and histograms are ±SD. Download Figure 1-4, TIF file.

10.1523/ENEURO.0057-21.2021.f1-5 Extended Data Figure 1-5 Validation of the semi-automated SPA script employing TrackMate.To validate the SPA software that was used for all dataanalysis, we employed a known dataset that was analyzed using the Metamorph plugin palmTracer. The data analyzed were derived from rat pheochromocytoma PC12 cells that were transfected with a Munc18-1mEos2 (Kasula et al., 2016)-tagged molecule. sptPALM was performed in the same way, except a lower exposure time of 20 ms was utilized. PC12 cells and Munc18-1mEos2 were provided by Frederic Meunier, Queensland Brain Institute. ***A***, The MSD of Munc18-1mEos2 trajectories and (***B***) AUC analysis reveals no significant difference between palmTracer and our custom TrackMate analysis scripts (*n *= 10, *p *=* *0.898, Wilcoxon matched pairs signed-rank test, 95% CI palmTracer 0.0142–0.0200, 95% CI TrackMate 0.0132–0.0213, MSD values presented as ±SD, AUC presented as ±5–95th percentile). Download Figure 1-5, TIF file.

Movie 1. Tracking individual Sx1a-mEos2 molecules in the fly brain. 10.1523/ENEURO.0057-21.2021.video.1

In order to characterize the mobility of individual tagged proteins, we performed SPT (Extended Data Fig. 1-1) as a *post hoc* step to image acquisition. We analyzed on average 2000–3000 individual trajectories of single Sx1a-mEos2 molecules over 16,000 frames (Fig. 1*H*) using the ImageJ software TrackMate (Tinevez et al., 2017) to localize molecules and perform particle tracking. Adapting TrackMate into a custom MATLAB interface, we analyzed the MSD (Fig. 1*I*) and molecule diffusion coefficients (Joensuu et al., 2017; Fig. 1*J*). On average, 10 molecules per frame were localized, with the majority of trajectories lasting 8 frames before terminating (Extended Data Fig. 1-4). We confirmed our analysis software by comparing our results with MSD data calculated using PALM-Tracer (a particle tracking plugin used in MetaMorph, Molecular Devices). Results were identical using either software (Extended Data Fig. 1-5).

To validate the reproducibility of our approach, we compared Sx1a-mEos2 mobility across successive recording sessions from the same brains. We recorded from different brain regions (Fig. 2*A–C*) and from the same brain region (Fig. 2*F–H*). We observed considerable variability in Sx1a-mEos2 mobility across experiments and brain regions (Fig. 2*D*,*E*), consistent with the large range in MSDs observed in our first dataset (Fig. 1*I*). Crucially, successive recordings from the same region (top right of the central brain, approximately in the lateral protocerebrum (Extended Data Fig. 1-3) revealed a high level of consistency in the number of localizations, trajectories, and MSD values within a recording site (Fig. 2*I*). This shows that results are repeatable in the same location, but also that some variability in diffusion coefficients exists across experiments in different brains (Fig. 2*J*). Importantly, successive recordings from the same brain region retained a similar number of localizations and trajectories, evident in highly comparable maximum projections of all the single molecule tracks (Fig. 2*G*,*H*) and the unchanged average spot and trajectory counts (Extended Data Fig. 2-1). We therefore proceeded with an internally controlled strategy centered on conditional neural activation in sequential recordings from the exact same location.

10.1523/ENEURO.0057-21.2021.f2-1 Extended Data Figure 2-1 ***A***, ***B***, Spot and trajectory counts between the first and second recording of Sx1a-mEos2 tracking experiments shows no difference in spot and trajectory counts (data from HL3.1 control recordings, *n *= 10, spot count *p *=* *0.9118, trajectory count *p *=* *0.7394, n.s., not significant. Statistics performed for both with a Wilcoxon test, data is ±5–95th percentile). Download Figure 2-1, TIF file.

### Conditional activation of brain neurons increases Sx1a mobility

Since the ionic composition of *Drosophila* extracellular fluid buffers varies in different experimental paradigms and can alter neuronal excitability (Feng et al., 2004), we examined the effects of different imaging solutions (Extended Data Fig. 3-1) and focused on HL3.1 buffer for all subsequent experiments. To ensure that the observed protein mobility was biologically relevant and not an artifact arising from the imaging solution, we performed the same experiment on brains that were first fixed in 4% PFA and then imaged in HL3.1 solution. Fixing the tissue resulted in a complete loss of Sx1a-mEos2 mobility (Extended Data Fig. 3-1; Movie 2). In addition to this, imaging only HL3.1 solution without any brain tissue revealed highly mobile bright spots that could be localized, but not tracked using our SPA software (Movie 3).

10.1523/ENEURO.0057-21.2021.f3-1 Extended Data Figure 3-1 Comparison of imaging buffers on the mobility of Sx1a-mEos2 particles in the *Drosophila* brain. During method development phase, several imaging buffers were trialed for physiological relevance and consistency between samples. Three random brain regions were sampled in UAS-dTRPA1>R57C10-Gal4 flies at 30°C for stimulation in either HL3, HL3.1, aCSF, or Schneider’s insect media and compared for their consistency. Also included is a mobility control where brains were fixed in a 4% PFA before imaging in HL3.1 solution, to confirm that tracked molecules are not an artefact of the imaging buffer. ***A***,MSD curves for the average of the three imaging buffers utilized with (***B***) the AUC highlighting a significant difference between HL3 to aCSF (HL3 *n *= 6, *p *=* *0.0058, AUC CI 0.0113–0.0160, HL3.1 *n *= 6, *p *=* *0.0316, AUC CI 0.0137–0.0161, aCSF*n *= 7, AUC CI 0.0165–0.0181, Schneider’s *n *= 4, *p > *0.999, AUC CI 0.01383–0.01882, Kruskal–Wallis test, MSD data presented as ±SD, AUC data presented as ±5–95th percentile). Despite aCSF providing the best consistency, HL3.1 was selected for its physiological relevance to *Drosophila* while retaining a degree of consistency above HL3. All imaging buffers were significantly different to the 4% PFA fixed brains, which showed minimal mobility effects (*n *= 6, *p *=* *0.0212 HL3, *p *=* *0.0067 HL3.1, *p *<* *0.0001 aCSF, *p *=* *0.0116 Schneider’s, AUC CI 0.00174–0.00478, Kruskal–Wallis test). Download Figure 3-1, TIF file.

Movie 2. Absence of Sx1a-mEos2 mobility in fixed tissue. 10.1523/ENEURO.0057-21.2021.video.2

Movie 3. Absence of Sx1A-mEos2 tracking in HL3.1 solution. 10.1523/ENEURO.0057-21.2021.video.3

Movie 4. Conditional paralysis at 30°C in w1118;Sx1a-mEos2/+;UAS-dTrpA1/R57C10-Gal4 flies and lack of paralysis at 30°C in w1118;Sx1a-mEos2/+;UAS-dTrpA1/+ controls. 10.1523/ENEURO.0057-21.2021.video.4

We next investigated whether we could increase Sx1a-mEos2 mobility when we stimulated neurons. In previous work, we have shown that Sx1a-mEos2 mobility increases on stimulation of larval motor nerve terminals, most likely because of the recruitment of Sx1a molecules to sites of active zones to form SNARE complexes, and that sustained activation of dTrpA1 channels leads to a consistent increase in spontaneous miniature junction potential frequency (Bademosi et al., 2016). To stimulate neurons in the adult fly brain, we employed a temperature-sensitive *Drosophila* transient receptor potential cation channel 1a (dTrpA1; Fig. 3*A*), which we expressed under UAS control using the pan-neuronal driver R57C10-Gal4 (Jenett et al., 2012), thereby allowing co-expression with Sx1a-mEos2. Conditional activation of dTrpA1 at 30°C from a baseline of 25°C allowed internally controlled experiments to be performed on the same recording site in the brain (Fig. 3*B*). Thus, all neuronal stimulation data could be normalized to the 25°C unstimulated condition at that recording site, thereby controlling for the variability observed across recording sites (Extended Data Fig. 3-2). To address potential drift in the tissue sample, we performed a cross-correlation analysis on the maximum projection data before and after dTrpA1 stimulation (Extended Data Fig. 3-3). We observed a consistent and significant increase in Sx1a-mEos2 mobility following thermogenetic stimulation, compared with baseline unstimulated conditions (*n *= 13, *p* = 0.0002, Wilcoxon test; Fig. 3*C*,*D*). In contrast, no significant increase in Sx1a-mEos2 mobility was observed at the elevated temperature in control brains that did not express dTrpA1 (Fig. 3*E*,*F*).

10.1523/ENEURO.0057-21.2021.f3-2 Extended Data Figure 3-2 Normalization of neuronal stimulation MSD curves to baseline. ***A***, Raw and average MSD curves for Sx1a-mEos2 recorded in the adult fly brain at 25°C, each color represents a different brain. ***B***, Raw and average MSD curves in the same brains as ***A***, but at 30°C for an internally controlled paradigm. Note high variance among brains (different colors) but low variance within (same colors). ***C***, Normalized MSD curve for the raw data in ***A***. The peak value of the curve at time point 0.30 (s) was used to normalize each time point, such that the peak of the normalized MSD curve at time 0.30 s is 1.0. ***D***, Normalized MSD curve for the raw data in ***B***, relative to within-brain baseline. Each time point in ***B*** was normalized to the matched peak value of the corresponding baseline curves in ***A***. All average data is presented as ±SD. Download Figure 3-2, TIF file.

10.1523/ENEURO.0057-21.2021.f3-3 Extended Data Figure 3-3 Correlation between Sx1a-mEos2 mobility and imaging region stability. Dual color images of Sx1a-mEos2 in a single brain comparing the initial 25°C (green) and second 30°C (red) recordings, showing the degree of overlap (gray) in a brain that (***A***) experienced drift and (***B***) experienced minimal to no drift. ***C***, ***D***, A Pearson correlation was calculated for 22 brains from the HL3.1 + DMSO condition, showing scatterplots for the degree of pixel correlation between the respective brains in ***A***,***B***. The pixel intensity of the green and red images in ***A***, ***B*** are plotted against one another with the brightness of the correlation scatterplot indicating the degree of overlap between each individual pixel. The four quadrants of the Cartesian plot indicate the distribution of pixel intensity for both the green and red images, with the top right quadrant indicating pixels that are highly correlated and the bottom left quadrant indicating pixels that highly uncorrelated. Regression line is shown. ***E***, Plotting the Pearson coefficients against the change in mobility reveals a correlation between an increase in Sx1a-mEos mobility with TrpA1 stimulation and low drift, whereas brains that drifted have a lower detectable Sx1a-mEos mobility (*n *= 22, slope = 0.6055, *R*^2^ = 0.3137, *p *=* *0.0067, solid line indicates best line of fit, dotted lines indicate 95% CI). Download Figure 3-3, TIF file.

### General anesthetics restrict Sx1a mobility in brain neurons

Having conditionally increased Sx1a-mEos2 mobility in the fly brain, we next sought to pharmacologically perturb this effect in the same preparation. We have previously shown that the intravenous general anesthetics propofol and etomidate decrease Sx1a-mEos2 mobility in mammalian neurosecretory cells as well as in *Drosophila* motor nerve terminals, by clustering Sx1a molecules on the presynaptic membrane (Bademosi et al., 2018b; Fig. 4*A*). Importantly, immobilization of Sx1a by propofol required a SNARE interaction domain; without this domain, propofol instead increased Sx1a mobility, as might be predicted because of increased membrane fluidity (Bahri et al., 2005, 2007). We therefore next investigated whether intravenous general anesthetics also decreased Sx1a-mEos2 mobility in the adult *Drosophila* brain, employing our internally controlled strategy. Consistent with our previous findings in other systems (Bademosi et al., 2018b), we found that 3 μm propofol and 8 μm etomidate impaired Sx1a-mEos2 mobility in fly brain neurons (Fig. 4*B*,*D*). Also consistent with previous work in mammalian cells and fly larvae (Herring et al., 2011; Bademosi et al., 2018b), an analog of propofol failed to restrict Sx1a-mEos2 mobility in the adult fly brain (Extended Data Fig. 4-1). We then proceeded to test other general anesthetics, to see whether different categories of drugs also had this immobilizing effect on Sx1a. In contrast to propofol and etomidate, the NMDA-acting sedative ketamine (100 μm) did not affect Sx1a-mEos2 mobility (Fig. 4*C*,*D*). We next tested two volatile drugs, isoflurane (0.19 mm) and sevoflurane (0.38 mm), and found that only isoflurane significantly impaired Sx1A-mEos2 mobility (Fig. 4*C*,*D*). We chose these concentrations approximating equipotency: the corresponding concentrations of isoflurane and sevoflurane in air (∼0.4% and ∼0.8%, respectively) both achieve significant behavioral effects in fruit flies (Zalucki et al., 2015; Olufs et al., 2018). The effect of isoflurane on Sx1a mobility was large enough to be evident even without requiring normalization (see non-normalized isoflurane data compared with propofol in Extended Data Fig. 4-2). In the clinic, propofol and sevoflurane are often used sequentially to maintain general anesthesia during surgery (Harris et al., 2006). We therefore questioned whether these intravenous and volatile drugs might have an additive effect on Sx1a mobility. Combining propofol with sevoflurane again significantly impaired Sx1a-mEos2 mobility, although not more so than propofol alone (Fig. 4*B*,*D*). Taken together, our anesthesia results show that the adult fly brain provides a physiologically relevant platform to study the effect of different drugs on single-molecule behavior in intact neural tissue. We show that Sx1a is highly dynamic in the adult fly brain, with increased mobility following neural stimulation and decreased mobility under exposure to certain general anesthetics. This confirms and expands findings in other model systems (Bademosi et al., 2016, 2018b), and shows that some commonly used intravenous and volatile general anesthetics might be affecting Sx1a mobility in the same manner. Importantly, we show the same effect for volatile as well as intravenous anesthetics, and that isoflurane in particular seems to have the greatest impact on Sx1a mobility.

10.1523/ENEURO.0057-21.2021.f4-1 Extended Data Figure 4-1 A structural propofol analog is not able to restrict Sx1a-mEos2 mobility. ***A***, Structure of the non-anesthetic analog of propofol (2,4-diisopropylphenol). Note the change in position of the hydroxyl group on the benzene ring from carbon 1 to carbon 3. ***B***,Under stimulation conditions, the MSD of Sx1a-mEos2 not able to be restricted in the presence of 3 μm of the propofol analog (orange) when compared to DMSO control (black), with no significant change in the (***C***)AUC (*n *= 8, *p *=* *0.9866, AUC CI 0.931–1.012, Kruskal–Wallis test, data for MSD is ±SD, data for AUC is ±5–95th percentile). Download Figure 4-1, TIF file.

10.1523/ENEURO.0057-21.2021.f4-2 Extended Data Figure 4-2 Non-normalized MSD and diffusion coefficients for Sx1a-mEos2 under propofol and isoflurane. ***A***, Raw MSD and AUC values for Sx1a-mEos under propofol (3 μm) at 25°C (baseline) and 30°C (TrpA1 stimulation) and (***B***) diffusion coefficients with corresponding mobile-to-immobile ratio (n.s., not significant, Wilcoxon test, paired statistics). ***C***, Raw MSD and AUC values for Sx1a-mEos in TrpA1 stimulated brains (30°C) with and without propofol. ***D***, Diffusion coefficients with corresponding mobile-to-immobile ratio (n.s., not significant, Mann–Whitney test, unpaired statistics). ***E***, Raw MSD and AUC values for Sx1a-mEos under isoflurane (0.19 mm) at 25°C (baseline) and 30°C (TrpA1 stimulation) and (***F***) diffusion coefficients with corresponding mobile-to-immobile ratio (*p < 0.05, Wilcoxon test, paired statistics). ***G***, Raw MSD and AUC values for Sx1a-mEos in TrpA1 stimulated brains (30°C) with and without isoflurane. ***H***, Diffusion coefficients with corresponding mobile-to-immobile ratio (n.s., not significant, Mann–Whitney test, unpaired statistics). Data are the same as normalized propofol and isoflurane data shown in Figure 4. Download Figure 4-2, TIF file.

In conclusion, we have shown that single mEos-tagged molecules can be resolved and tracked in the *ex vivo* brains of adult *Drosophila* fruit flies. This provides a useful and versatile tool for *Drosophila* researchers and those looking to perform super-resolution imaging of intact tissue, expanding on earlier inroads in this direction (Chen et al., 2014; Mir et al., 2018; Reisser et al., 2018). By employing an internally controlled paradigm, we were able to reliably increase the mobility of a presynaptic protein, Sx1a, through thermogenetic stimulation and restrict this mobility with the use of common general anesthetics. One caveat of our anesthetic results is that final concentrations in the brain tissue were approximated, based on previous experiments in other preparations. It remains possible, for example, that higher concentrations of sevoflurane or ketamine might also impair Sx1a mobility. Nevertheless, tracking single molecule dynamics in the *ex vivo* brains of adult *Drosophila* flies opens a new window into understanding the behavior of individual molecules in intact tissue, to, for example, help determine which mechanisms are drug-specific and which might reflect a common property of diverse drugs. Our results indicate that general anesthetics such as propofol and isoflurane might have similar effects among different kinds of chemical synapses. Although the adult fly brain is mostly cholinergic (Yasuyama and Salvaterra, 1999), we most likely sampled a variety of synapse types, including inhibitory synapses. That we found the same basic result (decreased Sx1a mobility) as in purely glutamatergic larval neuromuscular synapses (Bademosi et al., 2018b) argues for a common mechanism. Although our results focus on a ubiquitous presynaptic protein expressed in all neurons, the capacity to address circuit-specific questions could be expanded by adapting this approach to promoter-driven expression systems such as UAS/Gal4 on any protein target of interest. We believe this will result in highly reproducible and less variable results, as evidenced by the robustness of Sx1a-mEos2 mobility when recording in the same location twice. It will be interesting to apply SPT to investigate, for example, if Sx1a is equally compromised at excitatory versus inhibitory synapses, or to examine the individual dynamics of other proteins under general anesthesia, such as receptors in dedicated sleep/wake circuits in the fly brain (Kottler et al., 2013; van Swinderen and Kottler, 2014). Finally, a major advantage of conducting this work in animal models such as *Drosophila* is the capacity to efficiently test behavioral relevance, for example, as a way to relate local effects at the synapse with higher order behavioral readouts in behaving animals (Zalucki et al., 2015; Troup et al., 2019; van Swinderen and Hines, 2020).

## Synthesis

Reviewing Editor: Niraj Desai, NINDS Decisions are customarily a result of the Reviewing Editor and the peer reviewers coming together and discussing their recommendations until a consensus is reached. When revisions are invited, a fact-based synthesis statement explaining their decision and outlining what is needed to prepare a revision will be listed below. The following reviewer(s) agreed to reveal their identity: Roderic Eckenhoff, Misha Perouansky.

Your manuscript has been read by two expert reviewers and by me. We all were impressed by the sophisticated methodology and analysis, and we agreed that their application to the problem of syntaxin mobility in the fly brain would be of interest to many of eNeuro’s readers. Even so, the reviewers had a number of questions about the manuscript and suggestions for strengthening it. These are detailed in the review reports, which I have appended to this message. Please respond point by point.

One common area of concern (or, perhaps, confusion) was the anesthetic concentrations employed. As the first reviewer notes, straight calculations using the numbers at the top of p. 4 result in concentrations far larger than those reported in the Results section. These numbers may have been for intermediate or stock solutions which were then diluted further, but some clarification is required here. Beyond that point, both reviewers questioned the relationship between the reported concentrations and clinically or behaviorally relevant concentrations. The manuscript offers two references (Zalucki et al., 2015 and Bademosi et al., 2018b) that get at this question, but given the reviewer reactions, clearly more justification and/or explication is necessary.

Two other points beyond the review reports: (1) Please add a visual abstract to your resubmission. Visual abstracts help readers understand how to approach a manuscript. (2) Please include your code in your resubmission. It was not part of the Extended Data of the present submission and the URL was redacted for the double-blind review.

REVIEW #1

Summary: The authors provide a study of syntaxin mobility in fly brains using super-resolution microscopy. They show the predicted increase in motility with neuronal activation, and then ask whether various general anesthetics alter mobility as a potential explanation for decrease in neurotransmission that might contribute to anesthesia. In general, most anesthetics tested decreased syntaxin mobility, although ketamine and sevoflurane less so. What they cite as a non-anesthetic variant of propofol also has no significant effect on mobility.

Comments:

This is a very sophisticated examination of a reasonable hypothesis for a presynaptic contribution to the anesthetic endpoint in the fly. I have a few issues that need to be addressed before this can be considered for publication.

1. The anesthetic concentrations are confusing. On page 4 under “anesthetics", it says that 3uL isoflurane and 6 uL sevoflurane were dissolved into 20 mL HL3.1 solution. My calculations indicate such solutions are 1.2 and 2.2 mM - not the 19 and 38 uM indicated in the result section. (Both these drugs have a density of 1.5, meaning that 3 and 6 uL is 4.5 and 9 mg, MW of iso is 185 (sevo is 200), and with 20mL buffer, etc). So if these solutions were used straight, the concentrations were about 100-fold higher than indicated, or about 10-fold higher than clinical EC50 concentrations. If diluted to yield the indicated concentrations - why so low? 19 and 38 uM are about 10-fold lower than EC50. Especially considering that the propofol is about 2-fold higher than EC50 and ketamine about right. All these drugs should be compared at equipotent concentrations.

2. The non-anesthetic indicated has, to my knowledge, not been characterized as such. The reference cited is to the authors own work, that in turn cited an older paper as the source of this information. Checking this older paper shows that this particular compound was not tested. Please clarify.

3. The proposed mechanism underlying the decrease in syntaxin mobility is lateral diffusion. Are their data indicating that propofol and isoflurane decrease lateral diffusion at these concentrations? Most data suggest these drugs produce an increase in membrane fluidity which might be expected to enhance lateral diffusion.

REVIEW #2

Tracking single molecule dynamics in the adult Drosophila brain.

This work originates in a lab with an extensive track record of work in Drosophila, some experience with research in anesthetics and a publication record with respect to super resolution microscopy (the double blinding strategy is not very effective). As indicated to the editor, this reviewer has expertise in the first two categories but none in the third. Therefore I cannot evaluate the approach / analysis of the imaging data.

Overall, I think this work is innovative and I have only a few minor requests / comments.

General - what is known / documented about the duration / degree of viability of the dM brain in solution? Have ‘eeg’ recordings been made that support continued physiological activity?

L81: comparing propofol effects in the NMJ to the brain. NMJ transmitter is glutamate in the fly. What transmitter(s) is most prevalent in the D.m. and is likely photo-activated in your experiments? Is it known whether this (these) transmitters also rely on a SNARE complex with the same subunits / binding partners?

L110: why were females chosen?

L190: 16,000 frames at 30 msec is about 48 sec. What is known about deactivation kinetics of dTRPA1? What is the evidence that you actually depolarize nerve endings as opposed to a total prolonged depolarization of the neuron body which would result in seizure-like activity followed by ephys silence if you were to record an EEG?

What do you know about actual transmitter release during this massive, unspecific depolarization? Is it possible that the molecular movements that you observe do not relate to actual transmitter release?

L235 please define MSD at first use a couple of lines earlier.

L365 does expression of the R57C10-Gal4 driver result in a phenotype?

L397 while it is correct that isoflurane is the more potent agent, you apply sevoflurane at twice the concentration, hence your treatments are equipotent and the difference in effect is very interesting but not related to potency. Your referral to ‘potency’ appears misleading. In fact, a qualitative difference between two agents that at equipotent concentrations induce indistinguishable states of anesthesia suggests that these observations regarding molecular mobility have only limited significance for the state of general anesthesia.

Furthermore, please place the used concentrations (19 and 38 microM) into a behavioral context. What behavioral effects do you expect from such low (even by Dm standards) concentrations? Is there any reason to suspect that the pharmacologic properties of the SNARE complex differ between species?

L401 delivering simultaneously propofol and sevoflurane each one at anesthetic concentration is not a typical anesthetic. While both drugs are frequently used, they are ysed sequentially. During the ‘steady state’ of a vapor anesthetic, propofol is typically metabolized / redistributed.

## Author Response

Synthesis Statement for Author (Required):

Your manuscript has been read by two expert reviewers and by me. We all were impressed by the sophisticated methodology and analysis, and we agreed that their application to the problem of syntaxin mobility in the fly brain would be of interest to many of eNeuro’s readers. Even so, the reviewers had a number of questions about the manuscript and suggestions for strengthening it. These are detailed in the review reports, which I have appended to this message. Please respond point by point.

One common area of concern (or, perhaps, confusion) was the anesthetic concentrations employed. As the first reviewer notes, straight calculations using the numbers at the top of p. 4 result in concentrations far larger than those reported in the Results section. These numbers may have been for intermediate or stock solutions which were then diluted further, but some clarification is required here. Beyond that point, both reviewers questioned the relationship between the reported concentrations and clinically or behaviorally relevant concentrations. The manuscript offers two references (Zalucki et al., 2015 and Bademosi et al., 2018b) that get at this question, but given the reviewer reactions, clearly more justification and/or explication is necessary.

Two other points beyond the review reports: (1) Please add a visual abstract to your resubmission. Visual abstracts help readers understand how to approach a manuscript. (2) Please include your code in your resubmission. It was not part of the Extended Data of the present submission and the URL was redacted for the double-blind review.

REVIEW #1

Summary: The authors provide a study of syntaxin mobility in fly brains using super-resolution microscopy. They show the predicted increase in motility with neuronal activation, and then ask whether various general anesthetics alter mobility as a potential explanation for decrease in neurotransmission that might contribute to anesthesia. In general, most anesthetics tested decreased syntaxin mobility, although ketamine and sevoflurane less so. What they cite as a non-anesthetic variant of propofol also has no significant effect on mobility.

Comments:

This is a very sophisticated examination of a reasonable hypothesis for a presynaptic contribution to the anesthetic endpoint in the fly. I have a few issues that need to be addressed before this can be considered for publication.

1. The anesthetic concentrations are confusing. On page 4 under “anesthetics", it says that 3uL isoflurane and 6 uL sevoflurane were dissolved into 20 mL HL3.1 solution. My calculations indicate such solutions are 1.2 and 2.2 mM - not the 19 and 38 uM indicated in the result section. (Both these drugs have a density of 1.5, meaning that 3 and 6 uL is 4.5 and 9 mg, MW of iso is 185 (sevo is 200), and with 20mL buffer, etc). So if these solutions were used straight, the concentrations were about 100-fold higher than indicated, or about 10-fold higher than clinical EC50 concentrations. If diluted to yield the indicated concentrations - why so low? 19 and 38 uM are about 10-fold lower than EC50. Especially considering that the propofol is about 2-fold higher than EC50 and ketamine about right. All these drugs should be compared at equipotent concentrations.

We thank the reviewer for catching this mistake, as we have discovered an error in our reporting. The concentration of 3μL of isoflurane dissolved in 20mL is actually within the range of 0.15 to 0.23mM with an average of 0.19mM, not the 19μM we described here. This concentration was determined via repeated gas chromatography analysis of isoflurane samples in HL3 solution. These estimates are based off of previously published methods, which we reference (Sandstrom, 2004; Zalucki et al., 2015). We can provide these chromatography results, but they are available in Zalucki et al, 2015. We have checked that the calculations provided by the reviewer are correct and determined that any difference between the expected concentration (e.g., 1.2mM for isoflurane) and gas chromatography results (0.15-0.23mM) must be due to inevitable loss of isoflurane gas during the preparation. Nevertheless, we feel that 0.19mM isoflurane is a good approximation, and we are grateful that the reviewer caught this error. The volume for sevoflurane was selected to attempt to match an equipotent concentration of isoflurane, due to its oil/gas partition coefficient being approximately half of isoflurane (Alkire and Gorski, 2004). Interestingly with this result, twice the concentration compared to isoflurane was not able to produce a similar result. In fact, it was still half the mobility shift when compared to control. We have made the necessary amendments to the manuscript regarding isoflurane and sevoflurane concentration estimates in the methods, results, and discussion (lines 136-140, 406-408, 758-761, 941-944), clarifying that these are approximations.

Matching the anaesthetics concentration such that they are equipotent is difficult but is a valid concern particularly for mechanism-based experiments. In this study, we wanted to use previously determined concentrations of anaesthetics in similar assays to study the effects on syntaxin1A mobility as a proof of principle in order to validate our imaging paradigm (Sandstrom, 2004; Zalucki et al., 2015; Bademosi et al., 2018). We have amended the language in the text to better reflect this by making stronger references to previous work and highlighting the anaesthetic concentrations being approximate (lines 131-133, 408-412, 437-438).

2. The non-anesthetic indicated has, to my knowledge, not been characterized as such. The reference cited is to the authors own work, that in turn cited an older paper as the source of this information. Checking this older paper shows that this particular compound was not tested. Please clarify.

The reviewer is correct here that EC50 concentrations for 2,4-diisopropylphenol have not been characterised. We have used equimolar concentrations of this drug in conjunction with its anaesthetic counterpart, propofol (Bademosi et al., 2018). Others have used this propofol analog in mammalian cell culture assays, showing that twice the concentration of the propofol analog compared to propofol had no effect on the frequency of release events. We have now included this additional study as a reference for the analog, in the results in lines 401-403 (Herring et al., 2011), and have also removed the qualifier ‘non-anesthetic’ (line 402) because we agree that its effect on behavioural endpoints in animals remains unclear.

3. The proposed mechanism underlying the decrease in syntaxin mobility is lateral diffusion. Are their data indicating that propofol and isoflurane decrease lateral diffusion at these concentrations? Most data suggest these drugs produce an increase in membrane fluidity which might be expected to enhance lateral diffusion.

Correct, we believe that a specific drug-protein interaction is causing lateral trapping (which in turn, decreases lateral diffusion) of syntaxin1A molecules into nanoclusters. Whilst it appears paradoxical that such lipophilic drugs which usually increase membrane fluidity might have an opposite effect on syntaxin1A, we believe this argues for a specific anesthetic effect on the wild-type protein. We have shown in previous work that when the SNARE motif of syntaxin1A molecules is deleted, propofol actually has the opposite effect and increases the mobility of syntaxin1A, which is consistent with general increases in membrane fluidity and thus mobility of some proteins in the plasma membrane (Bademosi et al., 2018). This indicates to us that the anesthetic ‘trapping’ effects we observe on wild-type syntaxin1A depend on that protein’s interaction with other SNARE-associated proteins: when syntaxin1A cannot form SNAREs, it indeed moves faster under propofol exposure (Bademosi et al, 2018), as predicted by the reviewer. We now make this important point in our results, when we first refer to the previous propofol experiments (lines 390-394), and have also provided references for the effect of propofol on membrane fluidity (lines 394-397).

We are actively working on separate a mechanistic story, to understand specifically which other SNARE-associated proteins might be involved in this effect, and feel that the current result confirming impaired syntaxin1A mobility under diverse anesthetics is sufficient for concluding that similar effects might be happening in the adult fly central nervous system as at the larval NMJ.

REVIEW #2

Tracking single molecule dynamics in the adult Drosophila brain.

This work originates in a lab with an extensive track record of work in Drosophila, some experience with research in anesthetics and a publication record with respect to super resolution microscopy (the double blinding strategy is not very effective). As indicated to the editor, this reviewer has expertise in the first two categories but none in the third. Therefore I cannot evaluate the approach / analysis of the imaging data.

Overall, I think this work is innovative and I have only a few minor requests / comments.

General - what is known / documented about the duration / degree of viability of the dM brain in solution? Have ‘eeg’ recordings been made that support continued physiological activity?

This is a valid concern by the reviewer, as removing the brains from its physiological context is a damaging and stressful event. Ex vivo preparations of fly brains have a long history and remain a viable option to make informative electrophysiological recordings (e.g., see (Gu and O’Dowd, 2006)). This is currently the most common approach to address adult fly brain physiology (e.g., see (Raccuglia et al., 2019) which employed ex vivo brains of fruit flies to perform patch clamp and optogenetic voltage recordings over extended periods of time). Importantly, the authors of this last paper showed similar results between in vivo and ex vivo recordings, indicating the brains of these flies when dissected remain functional in extracellular fluid. Since we also see physiological effects of TrpA1 stimulation several minutes post-dissection (15-20 minutes), we are confident that the brains in this paradigm are viable for recordings. This nevertheless is a valid point and we have included these references into the discussion of our manuscript to make clear that ex vivo brain preparations are viable within the timeframe of our own experiments (approximately 25-30 minutes) (lines 296 -298).

L81: comparing propofol effects in the NMJ to the brain. NMJ transmitter is glutamate in the fly. What transmitter(s) is most prevalent in the D.m. and is likely photo-activated in your experiments? Is it known whether this (these) transmitters also rely on a SNARE complex with the same subunits / binding partners?

Fruit fly brains are predominately cholinergic (Yasuyama and Salvaterra, 1999) but of course also include a wide variety of other neurotransmitter systems. Since we focussed on the lateral protocerebrum, we are probably seeing a healthy mixture of different types of synapses in that part of the fly brain (Schultzhaus et al., 2017). SNARE mediated fusion is ubiquitous amongst most neuronal subtypes, with syntaxin1A/SNAP25/VAMP2 expression evident throughout the brain, so we are confident that what we are observing is a general effect on most synapses. Nevertheless, the reviewer brings up an important point that was perhaps overlooked: we see very similar effects of general anesthetics between (primarily) cholinergic synapses in the adult brain and glutamatergic synapses of the larval NMJ. We now highlight this point in the discussion (lines 447-449) and also provide a reference for the mostly cholinergic synapses of the adult fly brain (lines 445-447).

L110: why were females chosen?

Females were chosen to keep brains consistent amongst recordings. We also found them slightly easier to consistently dissect due to the heads of females being slightly bigger than males. We have included a sentence to explain our reason for focussing on females, in lines 150-151.

L190: 16,000 frames at 30 msec is about 48 sec. What is known about deactivation kinetics of dTRPA1? What is the evidence that you actually depolarize nerve endings as opposed to a total prolonged depolarization of the neuron body which would result in seizure-like activity followed by ephys silence if you were to record an EEG?

This is an interesting point and we have relied predominately on previous studies to make an assumption about neuronal depolarisation kinetics. Calculating 16,000 frames at 30 msec actually ends up being 8 minutes in total per recording, which is a much longer timeframe than what the reviewer questions. TrpA1 activation at 30{degree sign}C is quite robust and stable over time and only decreases when temperatures drop back to 25{degree sign}C (Bademosi et al., 2016). In that study, mEJP frequency was significantly increased and sustained over extended periods of TrpA1 stimulation in the larval neuromuscular junction, which indicates neuronal depolarisation. Since this stimulation protocol is almost identical to our own (although the tissue is admittedly different), we are confident that we are inducing neuronal depolarisation (activation). We have further clarified this in the main text (lines 367-371). Additionally, all of our flies were pre-screened behaviourally, to confirm that prolonged pan-neuronal activation of TrpA1 led to sustained seizure-like activity, as proposed by the reviewer. We show an example of this in our Supplemental Video 4. We feel that this behavioural evidence is convincing that neural activation is sustained throughout the 8min of our imaging experiments, although we concede that the exact kinetics remain undetermined.

What do you know about actual transmitter release during this massive, unspecific depolarization? Is it possible that the molecular movements that you observe do not relate to actual transmitter release?

Please see our answer to the related question, above. We should also add that behavioural recovery when flies are returned to 22{degree sign}C (room temperature) is quite rapid (minutes), again supporting our view that the imaging results reflect sustained neural depolarization.

Since the temperature is not a factor for syntaxin1A mobility in the absence of neuronal depolarisation (between 25{degree sign}C and 30{degree sign}C, Figure 3E,F of our manuscript), we believe that the increase in syntaxin1A diffusion must be physiologically relevant - i.e. activation of pre-synaptic neurons, which results in an increase in mEJP frequency, means that syntaxin1A molecules are being recruited to active zones out of nanoclusters. This is highlighted in lines 367-371.

L235 please define MSD at first use a couple of lines earlier.

Thank you for pointing this out, we have added the MSD definition at lines 225-229.

L365 does expression of the R57C10-Gal4 driver result in a phenotype?

This is not something we have tested. Usually, leaky expression of the UAS construct (in our case, UAS-TrpA1) is of concern for us hence our experiments in Figure 3E & F in which we did not drive the expression of TrpA1 with a Gal4. In a previous study, expression of the neuromuscular junction driver C380-Gal4 in the absence of UAS-TrpA1 had so significant effect on syntaxin1A mobility (Bademosi et al., 2016). We would expect that the reverse scenario, no UAS-TrpA1 present with R57C10-Gal4 expression, would result in the same phenotype.

L397 while it is correct that isoflurane is the more potent agent, you apply sevoflurane at twice the concentration, hence your treatments are equipotent and the difference in effect is very interesting but not related to potency. Your referral to ‘potency’ appears misleading. In fact, a qualitative difference between two agents that at equipotent concentrations induce indistinguishable states of anesthesia suggests that these observations regarding molecular mobility have only limited significance for the state of general anesthesia.

This is an excellent point and we have amended the language to indicate that we attempted to match the potencies of the two volatile anaesthetics by doubling sevoflurane’s concentration. These amendments have been made in lines 406-408.

Furthermore, please place the used concentrations (19 and 38 microM) into a behavioral context. What behavioral effects do you expect from such low (even by Dm standards) concentrations? Is there any reason to suspect that the pharmacologic properties of the SNARE complex differ between species?

Thank you for bringing this point up. As addressed in concern 1 from reviewer 1, we have reviewed our concentrations and realised we made an error. The concentrations of isoflurane and sevoflurane have been revised to be 0.19mM and 0.38mM respectively, which are estimates based upon previous chromatography experiments. Behavioural effects at equivalent concentrations of isoflurane have been observed (0.19mM in solution translated to approximately 0.4% in air) and include loss of coordination and responsiveness to vibrational stimuli (Sandstrom, 2004; Zalucki et al., 2015). Sevoflurane has also been studied in Drosophila behaviour, e.g., in relation to disease and toxicity, and has been shown to require approximately twice the concentration of isoflurane in air to achieve similar behavioural endpoints (Olufs et al., 2018). We have included this information in lines 408-412.

Since SNARE complex proteins are such highly conserved molecules across all species that express them, we have no reason to suspect any different pharmacological properties.

L401 delivering simultaneously propofol and sevoflurane each one at anesthetic concentration is not a typical anesthetic. While both drugs are frequently used, they are ysed sequentially. During the ’steady state’ of a vapor anesthetic, propofol is typically metabolized / redistributed.

This is correct, we apologise for the confusion. We have amended the text to make it more reflective of what occurs in a clinic (lines 414-417).

References

Alkire MT, Gorski LA (2004) Relative Amnesic Potency of Five Inhalational Anesthetics Follows the Meyer-Overton Rule. Anesthesiology 101:417-429 Available at: https://pubs.asahq.org/anesthesiology/article/101/2/417/9202/Relative-Amnesic-Potency-of-Five-Inhalational.

Bademosi AT, Lauwers E, Padmanabhan P, Odierna L, Chai YJ, Papadopulos A, Goodhill GJ, Verstreken P, van Swinderen B, Meunier FA (2016) In vivo single-molecule imaging of syntaxin1A reveals polyphosphoinositide- and activity-dependent trapping in presynaptic nanoclusters. Nat Commun 7:13660 Available at: http://www.nature.com/articles/ncomms13660.

Bademosi AT, Steeves J, Karunanithi S, Zalucki OH, Gormal RS, Liu S, Lauwers E, Verstreken P, Anggono V, Meunier FA, van Swinderen B (2018) Trapping of Syntaxin1a in Presynaptic Nanoclusters by a Clinically Relevant General Anesthetic. Cell Rep 22:427-440 Available at: https://linkinghub.elsevier.com/retrieve/pii/S2211124717318788.

Gu H, O’Dowd D (2006) Cholinergic Synaptic Transmission in Adult Drosophila Kenyon Cells In Situ. J Neurosci 26:265-272 Available at: http://www.jneurosci.org/cgi/doi/10.1523/JNEUROSCI.4109-05.2006.

Herring BE, McMillan K, Pike CM, Marks J, Fox AP, Xie Z (2011) Etomidate and propofol inhibit the neurotransmitter release machinery at different sites. J Physiol 589:1103-1115 Available at: http://doi.wiley.com/10.1113/jphysiol.2010.200964.

Olufs ZPG, Loewen CA, Ganetzky B, Wassarman DA, Perouansky M (2018) Genetic variability affects absolute and relative potencies and kinetics of the anesthetics isoflurane and sevoflurane in Drosophila melanogaster. Sci Rep 8:2348 Available at: http://www.nature.com/articles/s41598-018-20720-7.

Raccuglia D, Huang S, Ender A, Heim M-M, Laber D, Suárez-Grimalt R, Liotta A, Sigrist SJ, Geiger JRP, Owald D (2019) Network-Specific Synchronization of Electrical Slow-Wave Oscillations Regulates Sleep Drive in Drosophila. Curr Biol 29:3611-3621.e3 Available at: https://linkinghub.elsevier.com/retrieve/pii/S0960982219311200.

Sandstrom DJ (2004) Isoflurane depresses glutamate release by reducing neuronal excitability at the Drosophila neuromuscular junction. J Physiol 558:489-502 Available at: http://doi.wiley.com/10.1113/jphysiol.2004.065748.

Schultzhaus JN, Saleem S, Iftikhar H, Carney GE (2017) The role of the Drosophila lateral horn in olfactory information processing and behavioral response. J Insect Physiol 98:29-37 Available at: https://linkinghub.elsevier.com/retrieve/pii/S0022191016302736.

Yasuyama K, Salvaterra PM (1999) Localization of choline acetyltransferase-expressing neurons inDrosophila nervous system. Microsc Res Tech 45:65-79 Available at: https://onlinelibrary.wiley.com/doi/10.1002/(SICI)1097-0029(19990415)45:2%3C65::AID-JEMT2%3E3.0.CO;2-0.

Zalucki OH, Menon H, Kottler B, Faville R, Day R, Bademosi AT, Lavidis N, Karunanithi S, van Swinderen B (2015) Syntaxin1A-mediated Resistance and Hypersensitivity to Isoflurane in Drosophila melanogaster. Anesthesiology 122:1060-1074 Available at: https://insights.ovid.com/crossref?an=00000542-201505000-00021.
