# Supplementary material for: Tracking Single Molecule Dynamics in the Adult Drosophila Brain
Source: eNeuro. 2021 May 14;8(3):ENEURO.0057-21.2021. doi: 10.1523/ENEURO.0057-21.2021 (PMC8174007; doi:10.1523/ENEURO.0057-21.2021)
Supplement: Extended Data Document 1 — sptPALM analysis guide. Download Extended Data Document 1, DOCX file. [file enu-eN-NWR-0057-21-s06.docx]

sptPALM Analysis Guide


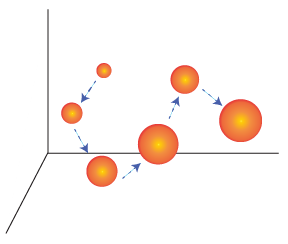


Adam Hines

A simplified particle localisation and tracking solution.

Contents

[1 Introduction 4](#_Toc9785)

[1.1 Overview 4](#_Toc9786)

[1.2 Spot detection 4](#_Toc9787)

[1.3 Particle linking 5](#_Toc9788)

[1.4 Mean squared displacement and diffusion coefficients 7](#_Toc9789)

[1.5 Comparison of SPA to MetaMorph/PALMTracer 9](#_Toc9790)

[2 Using SPA 11](#_Toc9791)

[2.1 Installing and setting up SPA 11](#_Toc9792)

[2.2 Preparation of image sequences 15](#_Toc9793)

[2.3 File directory structure 17](#_Toc9794)

[2.4 Setting analysis parameters 18](#_Toc9795)

[2.5 Setting threshold values 19](#_Toc9796)

[2.6 Running analysis and results 20](#_Toc9797)

[2.7 Troubleshooting 22](#_Toc9798)

[2.7.1 Java Heap Memory 22](#_Toc9799)

[2.7.2 Image files too large 22](#_Toc9800)

[3 Example Analysis 22](#_Toc9801)

# Introduction

Thank you for downloading and trying sptPALM Analysis (SPA). SPA is a semi-automated single particle tracking tool utilising a graphical user interface (GUI) that interfaces with the ImageJ plugin TrackMate. Users are able to automatically load image sequences of photoactivatable localisation microscopy (PALM) and utilise a built in tracking algorithm for single particles (spt). The semi-automated component refers to the fact that users are required to convert image sequences to the correct format and determine threshold values for spot detection themselves. This is advantageous as auto-detecting threshold values can often lead to over or under-estimation of the spot detection threshold.

SPA was developed by Adam Hines at the Queensland Brain Institute. If utilising this tool in your publications, please cite (*reference*) as well as the original author of TrackMate (*reference*).

## Overview

This chapter will provide an overview on how the analysis script operates and what algorithms the program uses in order to reliably detect and track the mobility of particles. It will begin by briefly describing the spot detection component of the analysis and the tracking algorithm utilised, however for further information please refer to the source documentation of TrackMate ([TrackMate Algorithms)](https://imagej.net/TrackMate_Algorithms).

## Spot detection

The chosen method for spot detection was the Lapacian of Gaussian (LoG) fitting algorithm. This algorithm takes low resolution spots and fits a LoG over pixels in an x,y plane. The threshold specified will determine which detected spots are appropriate and which are not, and this should be done by the user with their own eyes as this avoids having too many innappropriate or too few appropriate spots. A visualisation of how the spot detection algorithm works is presented in Figure 1.

There are also two settings that are enabled during the spot detection component of SPA. Median filtering and subpixel localisation are enabled to remove the generation of ’jagged’ particle linked tracks as well as to improve the acuity of spot detection.

There are other methods for spot detection available in TrackMate, and it should be encouraged to explore the best option for your needs. Embedded into the software however is the LoG detection. SPA is an open-sourced program, so editing the source code will be your only option in order to hardwire a difference detection algorithm - and the same goes for the tracking method (covered in the next section).


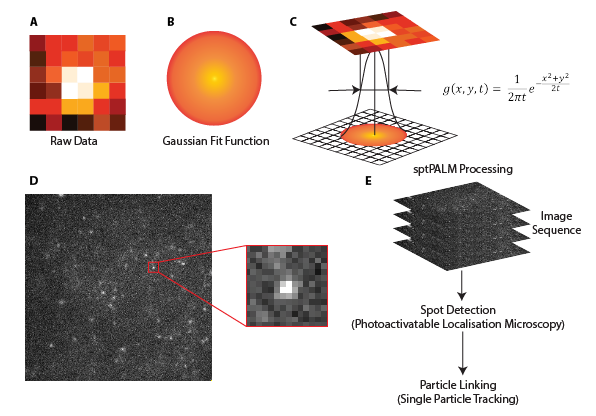


Figure 1: Visualisation of particle detection in SPA. **A** Schematic of typical raw data observed in photoactivatable light microscopy (PALM) experiments, consisting of low resolution pixels with a bright centre with decreasing intensity from the centre. **B** Example of a Gaussian fit of the raw data in **A**. **C** The Gaussian fit function transposing the low resolution raw data into a high-resolution spot. Formula shown if the Lapacian of Gaussian (LoG) spot detection used in SPA analysis. **D** Representative raw data from a PALM experiment highlighting a single spot. **E** Schematic of the workflow used in SPA. Image sequences have parallel threading in spot detection and particle linking in subsequent tracking stages of analysis.

## Particle linking

Single particle tracking has remained a problem for researchers for a very long time. Reliable tracking of multiple particles over a long time frame can cause issues such as linking particles together that are not the same, incorrectly linking a particle that has newly appeared, or even incorrectly ending a track. Figure 2 **A** shows an example of what a tracked particle looks like in an x,y plane. SPA uses the particle linking algorithm known as a linear assignment problem (LAP) cost matrix in order to evaluate and ultimately minimise the cost in favour of the most likely linked particles between frame *t*_1_ and *t*_2_. The cost values in this context is simply the squared displacement (*δ*^2^) between two detected particles. Each particle in frame *n*_1_ has a potential link associated to every particle in frame *t*_2_ (Figure 2 **B**), meaning that for each particle in *t*_1_ there are several cost values associated to that particle and the LAP algorithm attempts to find the lowest sum cost for every single particle between frames *t*_1_ and *t*_2_.

What about particles that have no prior or future link? At a certain point during a recording, particles will bleach and new particles will appear, so how are these dealt with? A separate matrix is established where the previous cost value of a particle linking, (*δ*^2^), is multiplied by a factor of 1.05. If there is a cost that is lower than this value, the particle will have a potential link. If the multiplied cost factor satisfies the lowest sum calculation then that particle, depending on context, will either have no future linkages or is defined as a newly appearing particle.

Particles that are at disparate ends of an x,y plane are easily dealt with by simply setting a maximum cutoff that two particles can physically link. If the (*δ*^2^) exceeds this value, then the link is impossible and cannot satisfy the lowest cost sum. A summary of the potential outcomes of a particle linking between two frames is shown in Figure 2 **C**.


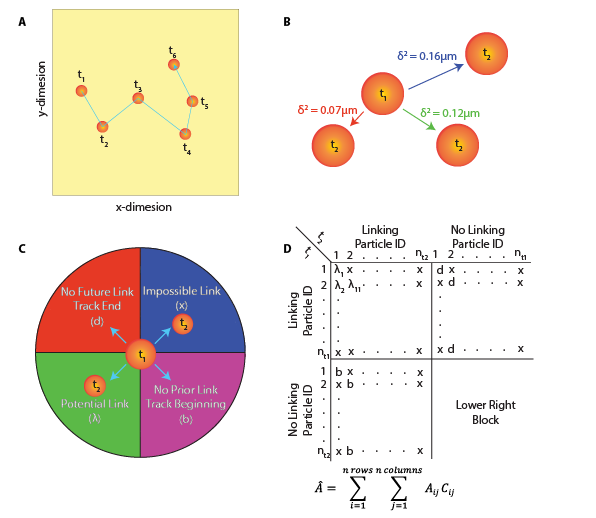


Figure 2: Use of a Linear Assignment Problem (LAP) Cost Matrix to Link Particles Between Frames. **A** Schematic of how a particles trajectory through time in an x,y plane may look, *t_n_* refers to the time or frame number. **B** A particle in *t*_1_ will have multiple potential linking particles in *t*_2_, with an associated cost value *δ*^2^. **C** The possible outcomes for a particle in *t*_2_ following *t*_1_. **D** Schematic of the cost matrix utilised in the LAP. To satisfy the LAP, the sum of the cost is minimised as low as possible.

## Mean squared displacement and diffusion coefficients

The main metric utilised in single particle tracking is the mean squared displacment (MSD), which describes the distance a particle moves from its initial displacement in relation to time. In association with the MSD, the diffusion coefficient is another metric that describes the diffusivity of molecules in space. The diffusion coefficient is empirically derived from the MSD and combined describe the mobility of tracked particles (Figure 3 **A** and **B**). The diffusion coefficient values can be represented on a curve as a relative frequency of a whole, taking a logarithmic conversion and counting the frequency in bins of -5 to 1.

Useful quantifications of the MSD and diffusion coefficients are the area under the curve and the mobile to immobile ratio, defined as the ratio between binned log diffusion coefficients above -1.6 (related to a speed of X) and below -1.6 (Figure 3 **C** and **D**). These two metrics allow for simple statistical analysis.

SPA automatically calculates the MSD and diffusion coefficients thanks to a piece of code from *Reference*. Area under the curve and mobile to immobile ratios are not currently calculated within SPA.


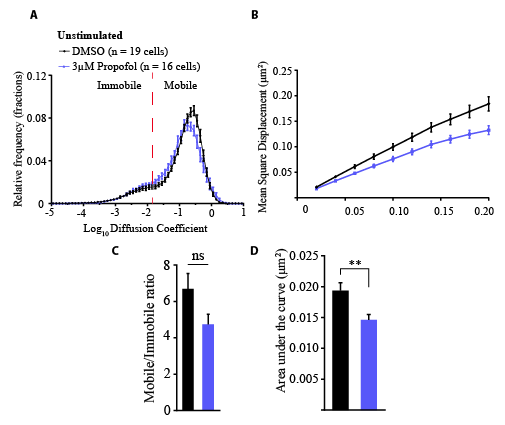


Figure 3: Example of the metrics of single particle tracking experiments. **A** Relative frequency of diffusion cofficients in Munc18-1 mEos2 tracked particles transfected in PC12 cells stimulated with *BaCl*_2_ in the presence of dimethyl sulfoxide (DMSO) or

3*µM* propofol. **B** Mean square displacment of mEos2 particles tracked over time. **C** Mobile to immobile ratio calculated from the diffusion coefficients in **A** and area under the curve calculated from **B**.

## Comparison of SPA to MetaMorph/PALMTracer

How does SPA stack up against other localisation and tracking software? Given that TrackMate is a completely open-sourced and free plugin that also happens to run in an open-source and free software, Fiji, how would it fare against MetaMorph - a several thousand dollar piece of software that is often unaccessible to a lot of researchers. MetaMorph makes use of a plugin called PALMTracer, developed by John-Baptiste (*Reference*), in order to localise and track particles. PALMTracer is not an automated workflow and requires the user to manually process each image sequence themselves, in comaprison to SPA which allows users to click a single button and walk away.

We can clearly see in Figure 4 that there is no significant difference between analysis performed on MetaMorph vs SPA/TrackMate.


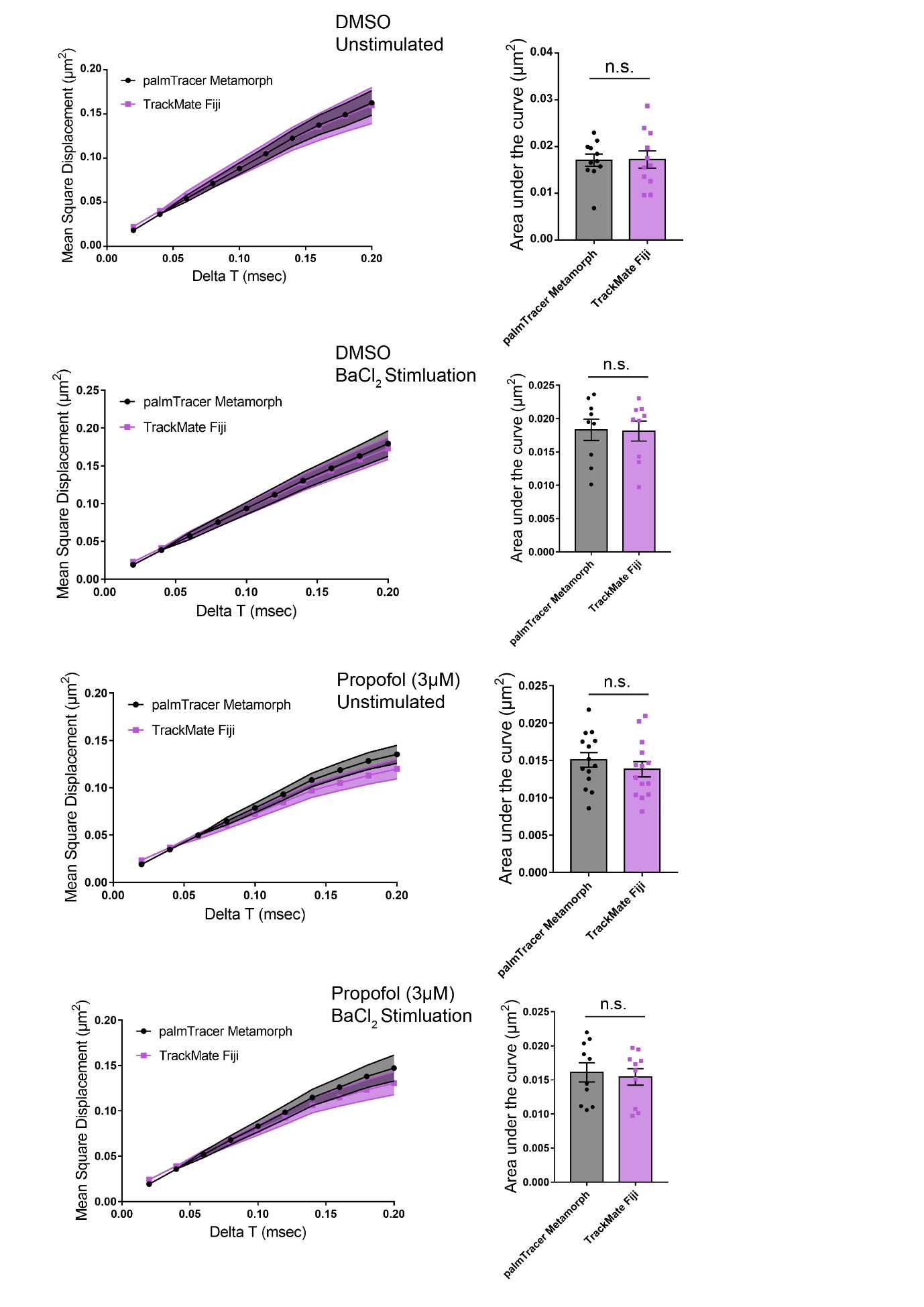


# Using SPA

This section will explore how to setup, run, and look at the output of SPA. In order to run SPA, you will require MATLAB 2017b or higher as well as some considerable computing power. SPA will run on most modern computers, however older machines may struggle to allocate appropriate computing power for analysis, particularly for large image sequences (1gb). There is a copy of Fiji included with the download and is stored locally in the analysis folders.

## Installing and setting up SPA

To begin, obtain the latest version of SPA through GitHub. Create a GitHub account and download the code and store it somewhere local on your computer (e.g. the MATLAB directory). Access the SPA GitHub repositry [here,](https://github.com/AdamDHines/sptPALM-Analysis.git) click ”Clone or download”, and then ”Download ZIP” (Figure 5).


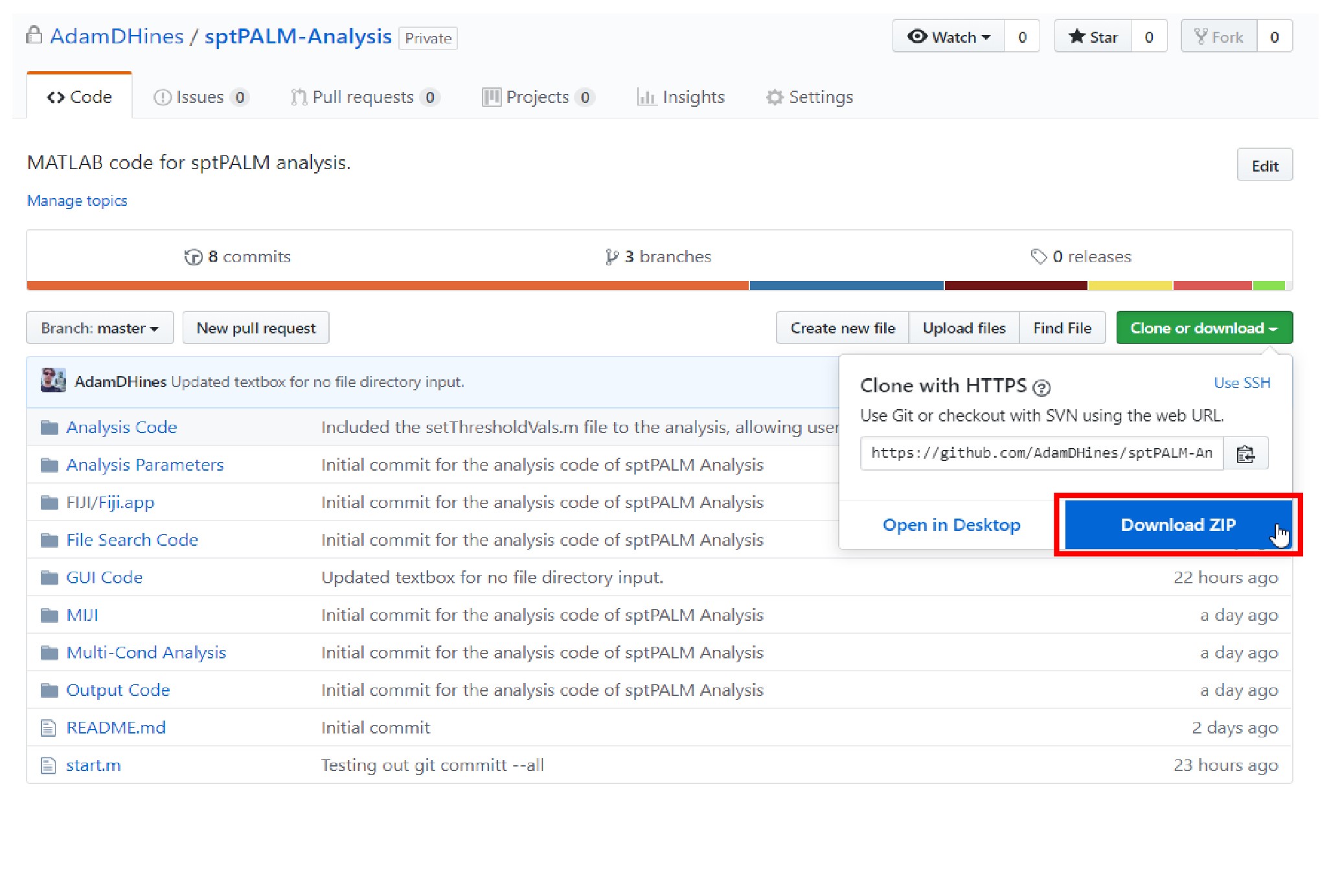


Figure 4: Accessing GitHub to download the latest version of SPA.

Once the ZIP folder has been downloaded, create a folder such as ”sptPALM” in a local directory (e.g. the MATLAB folder in Documents) and open MATLAB. Once open, you must set the current directory of MATLAB to the folder that you created that contains all of the code and the ”start.m” file. To do so, click the folder icon with the green arrow just above the Current Folder panel on the left hand side (Figure 6).


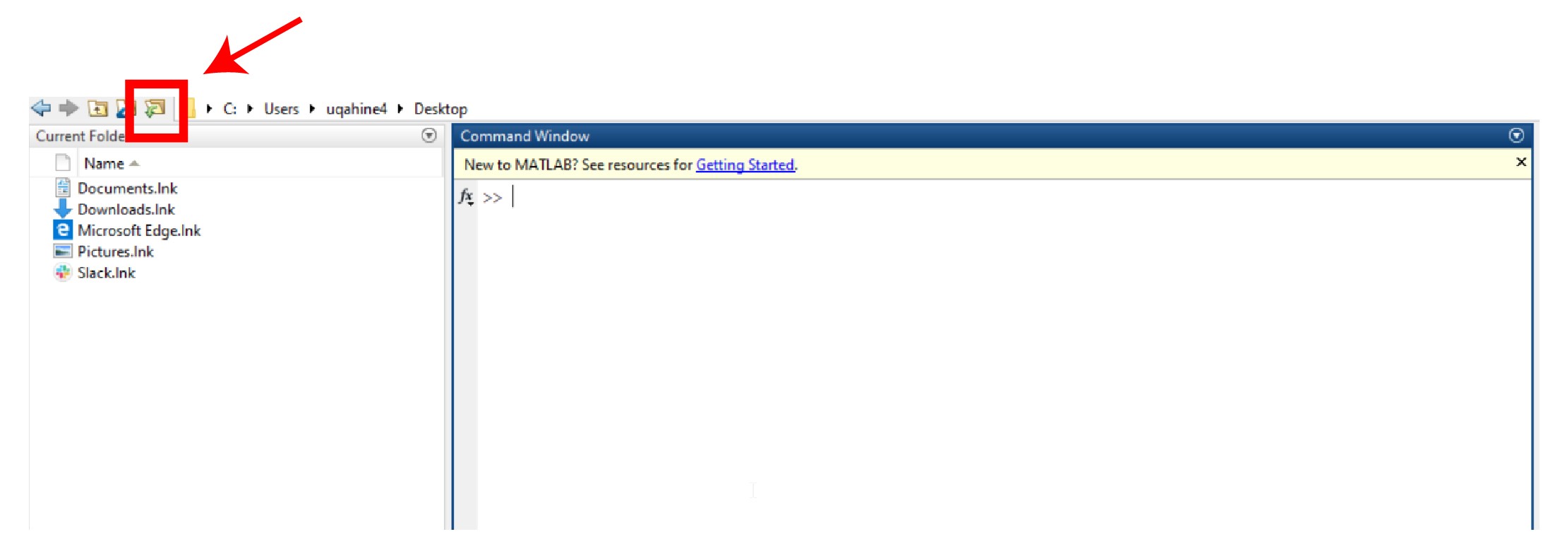


Figure 5: Changing the current working directory to the SPA analysis folder.

Once this is complete, your Current Folder in MATLAB will look something like this (Figure 6).

Now we can go ahead and run SPA. In the command window of MATLAB, type

”start” and hit enter. This will initialise the *start.m* script and launch the graphical user interface. When a fresh install of SPA is performed, you will need to set a default directory to search for files. This can be done by simply pressing ”Set directory” in the File directory panel up the top or by going File - Set File Directory (Figure 7). Once this is done, the default directory will be saved and you won’t have to set it again.

Congratulations, SPA is now setup on your machine. The next few sections will detail how data files should be prepared and organised in order for the analysis to work.


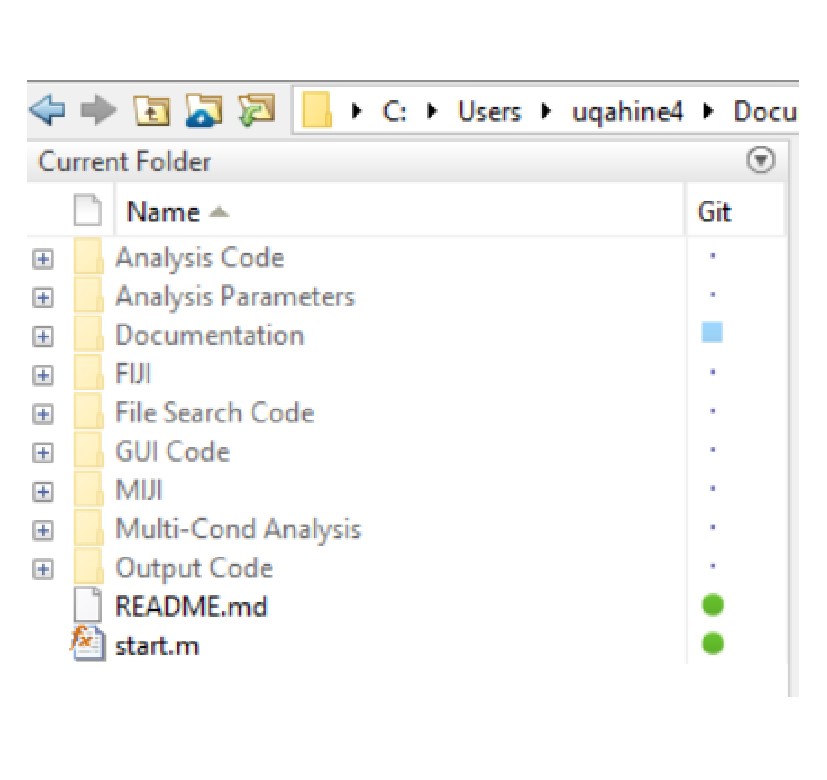


Figure 6: Current directory with SPA code.


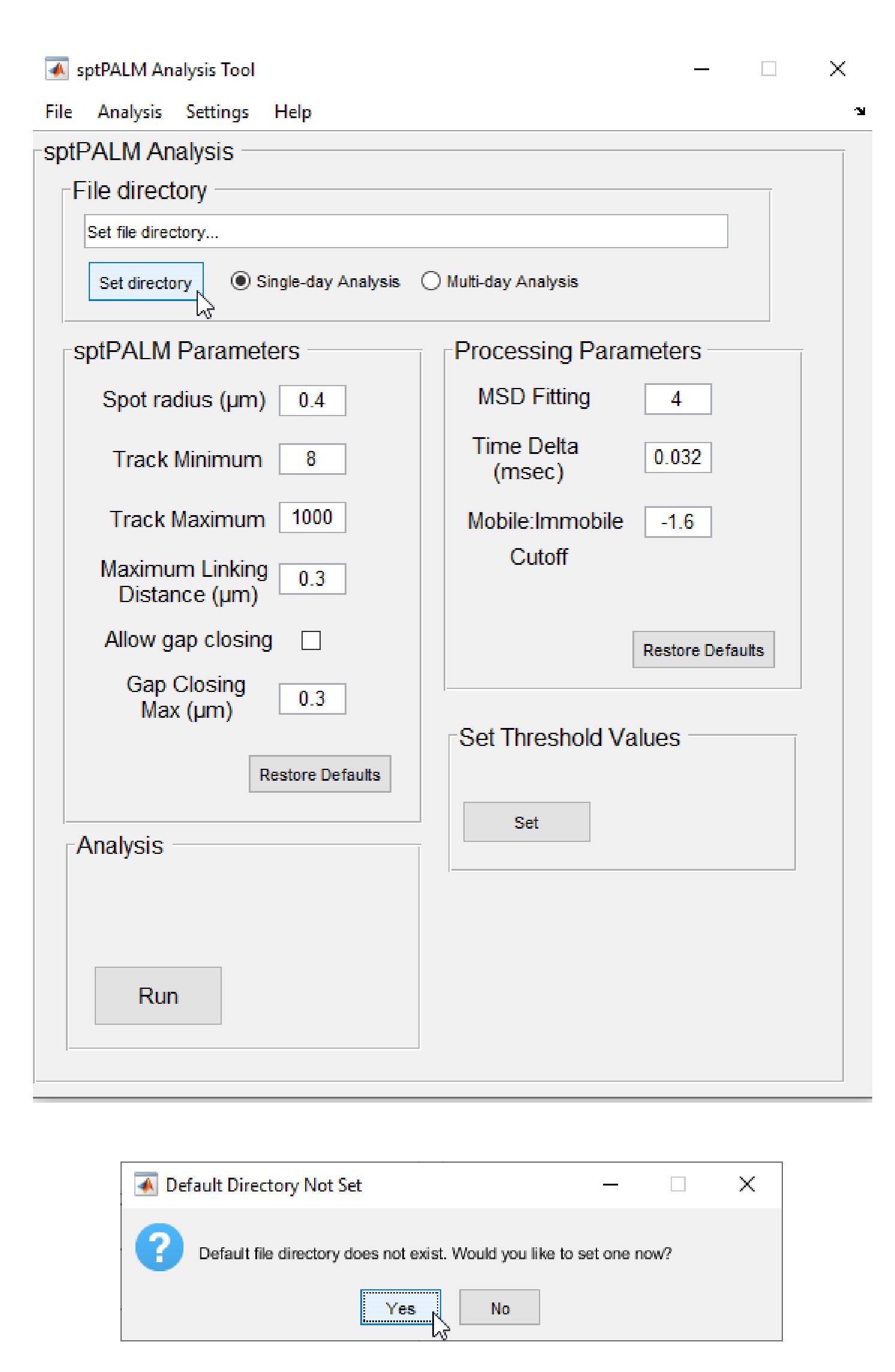


14

Figure 7: Setting the default working directory in SPA.

## Preparation of image sequences

The image sequences that are used in SPA are .tif stacks. Any other file will not be recognised by SPA and won’t be able to be utilised in analysis. Therefore, it is necessary to convert any image sequences to .tif using ImageJ. It is highly recommended that when exporting image sequences from the imaging software of your choice, that you save it in the native format in order to retain metadata about the recordings and then convert to .tif afterwards. What we will also do during this time is establish our threshold values for the spot detection component of the analysis.

To begin, open your image sequence in ImageJ. Check the sequence that the properties are correct, i.e. that the calibration settings for pixel height and width have been set and that the sequence is in terms of t and not z (analysis will stall if the t and z are swapped). Also ensure that the unit of length is ”micron”. Change the settings if necessary and hit ’OK’ (Figure 8). In general, if you’re loading the sequence from the source file containing the metadata ImageJ will automatically alter the properties to match.


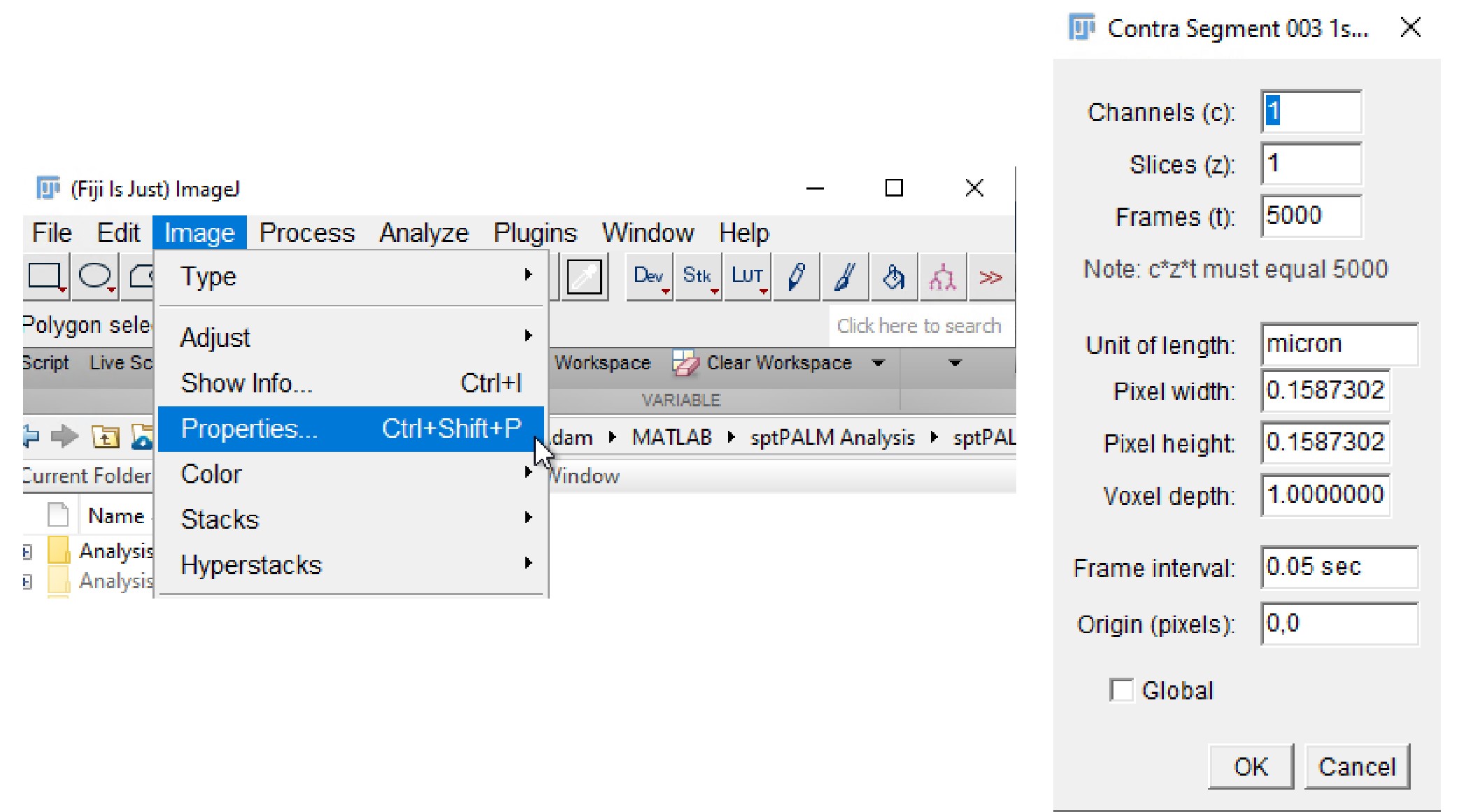


Figure 8: Checking the image sequence properties for calibration and correct z and t orientation.

We now want to determine the threshold value, as well as the spot diameter, for the localisation component of the analysis. Open TrackMate by clicking Plugins → Tracking → TrackMate. The TrackMate GUI will open and simply click next until you reach the ’LoG Detector’ settings panel. Here, determine the appropriate spot diameter and threshold value for the recording. *Important* Ensure that both median filtering and subpixel localisation are selected. Use the preview button to view how the settings localise spots. Make note of the diamaeter and the threshold value for each individual movie, we will tell SPA when we come to analyse the files what these are.


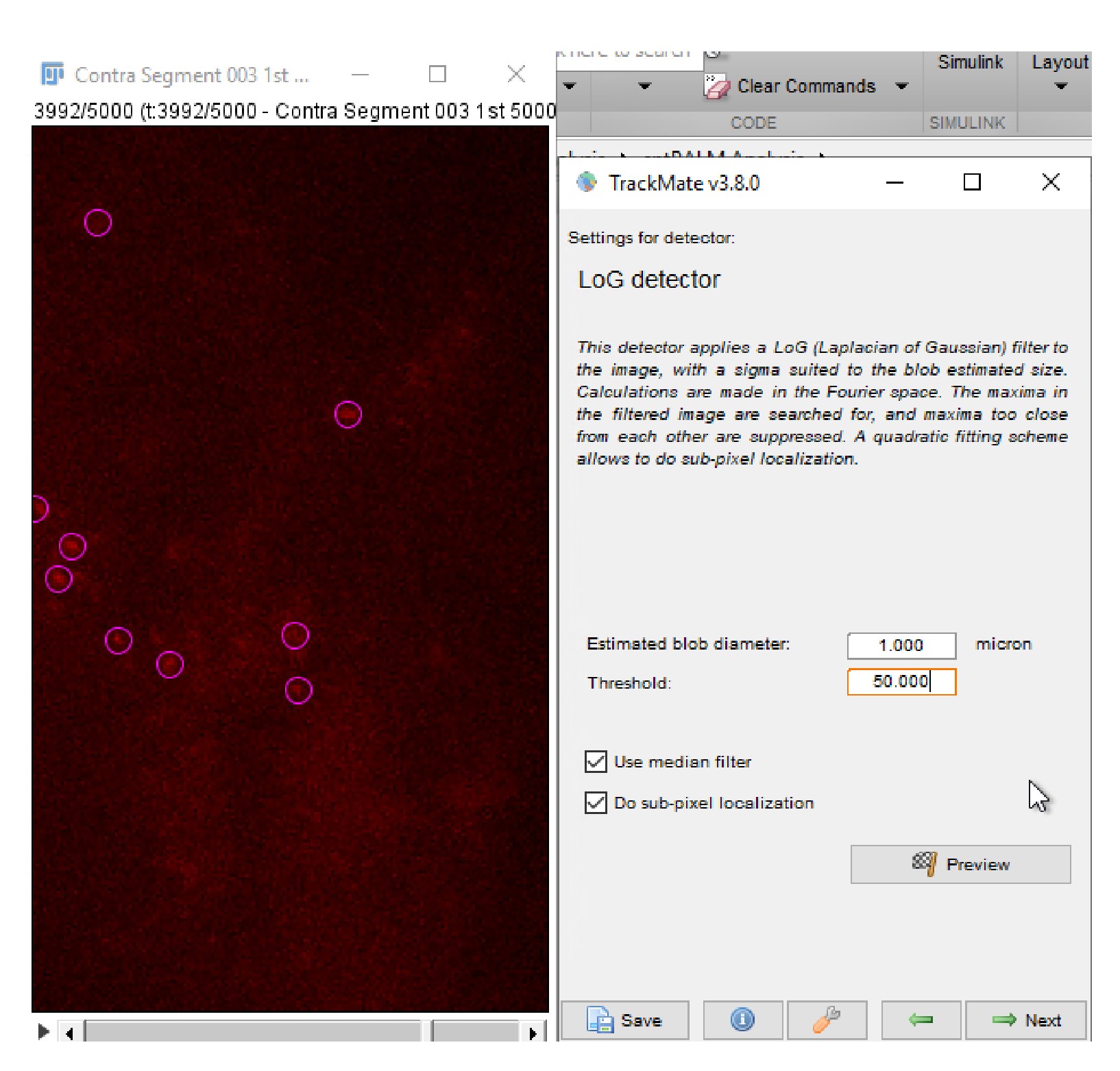


Figure 9: Setting the spot diameter and threshold value for image sequences.

Once that’s done, all we need to do now is save the sequence as .tif by going to File → Save as... → Tiff... The next section will show you how to organise your data structures such that the analysis script can search for and find the files for analysis.

## File directory structure

The file structure for the image sequences is very crucial and must be followed exactly. If it is not follow correctly, then the analysis script will not load or find any sequences to analyse and will just throw an error. In short, the sequences are all organised into individual folders inside of a parent folder. An example of this would be a parent folder of *Sx1a-mEos2 28-03-2019* and within this folder are several sub-folders *Recording 1, recording 2, recording 3, etc.*. The sub-folders are where the original recording (the one containing meta-data) and the .tif converted file should be stored. Adjusting to this file structure from the beginning from saving the initial recordings will save a lot of time in the future. *Important* Only store **1** .tif file in a subfolder, more than 1 .tif files in a subfolder will cause errors in the software.

Let’s have a look at what this file structure is. From left to right, we have the main data folder where we can see the folder *20190320* - the folder that contains the sub-folders with our recordings in them. In the middle, we see all of the sub-folders of individual recordings, and on the right we have the contents of the sub-folder. Notice the raw *.czi* file and the converted .tif that has been made. SPA will search through all of the folders and try to find a .tif file, and eliminate everything else. This is why it’s so important to convert all of the image sequences into .tif before continuing. If a .tif is missing from the folder, the analysis will still run but skip over that experiment.


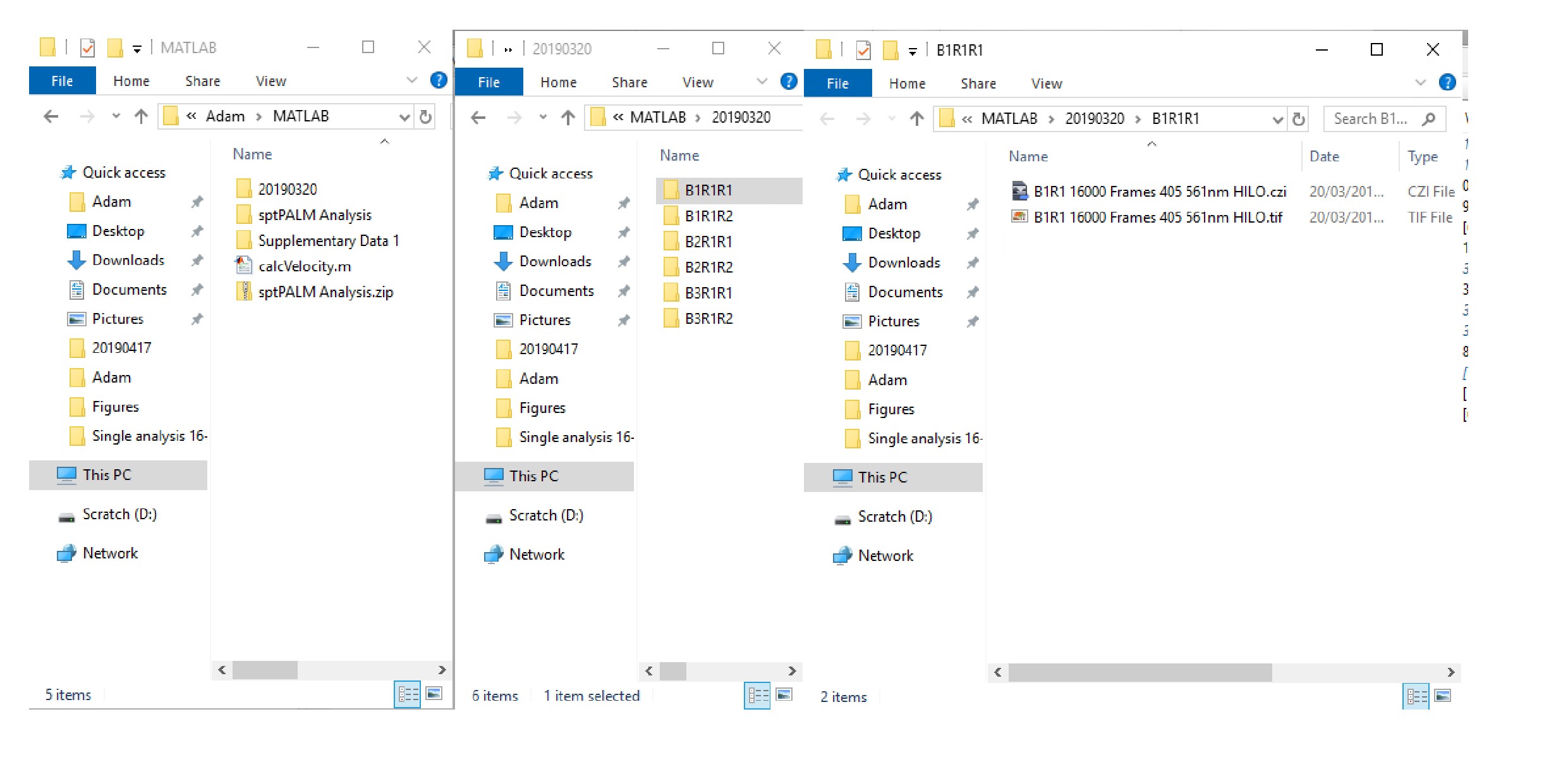


Figure 10: Folder directory structure for the analysis of files.

SPA will output all of the analysis into the same parent folder as the analysis, so getting used to this structure early on will be of great benefit to using the software. The next section will focus on how to set the analysis parameters (but not how to derive them to tailor your experiments).

If you wish to split your files out for analysis, i.e. splitting a 16,000 frame image sequence into 2,000 frame sequences for purposes of allowing analysis to run, please keep the same file directory format and select ”File Splitting?” when running analysis. This will simply ignore the 1,000 trajectory or greater rule that applies to analysing the MSD or diffusion coefficient. This button may also be used to simply ignore the rule all-together if required.

## Setting analysis parameters

Setting the analysis parameters is easy in SPA. If you wish to make a change to any of the analysis parameters, simply edit the text box with the new desired parameter (Figure 11).

- **A** Set the spot radius for localisation, note that this is half the ’blob diameter’ used in determining thresholds (section 2.2)
- **B** Set the minimum number of spots in a track for analysis, i.e. the minimum track length.
- **C** Set the maximum number of spots in a track for analysis, i.e. the maximum track length.
- **D** Set the maximal linking distance between two spots between frame *t*_1_ and *t*_2_.
- **E** Select checkbox if you wish to allow gap closing for disappearing spots, gap closing is disabled by default.
- **F** Sets the maximum gap closing distance if gap closing is selected.
- **H** Restore the default values for the sptPALM Parameters.
- **I** Number of points that will be used to fit the MSD for diffusion coefficients.
- **J** The time scale for image sequences, i.e. exposure time between frames.
- **K** Sets the mobile:immobile ratio cutoff, by default this is -1.6.
- **L** Restore the default values for the Processing parameters.
- **N** Select whether or not original movie files are split (ignoring the 1,000 trajectory minimum rule), file splitting disabled by default.


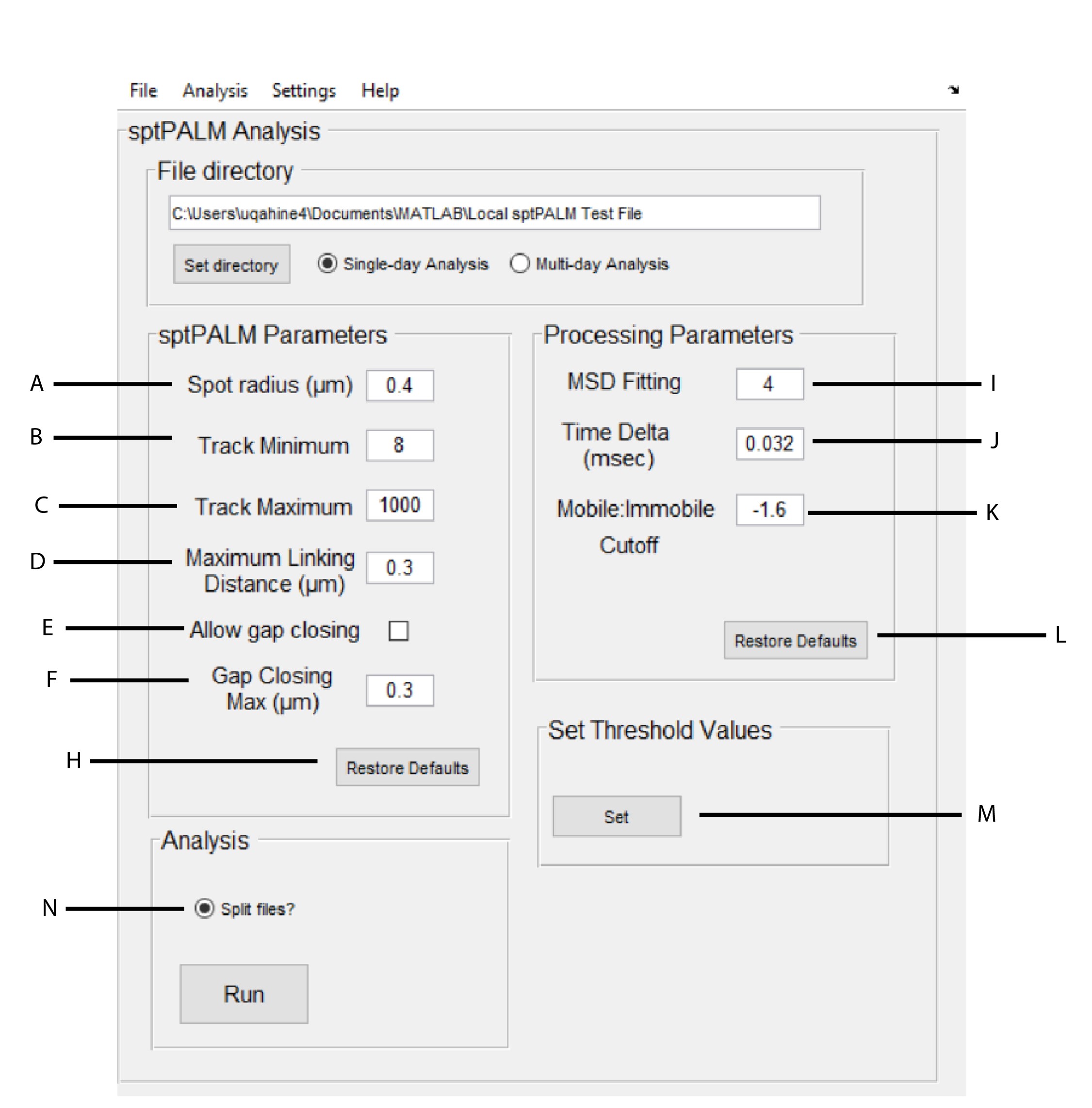


Figure 11: Analysis parameters for SPA.

## Setting threshold values

The threshold values determined in the TrackMate GUI can now be directly input into the SPA GUI. When a file directory is selected, click the Set button in the ”Set Threshold Values” panel (**M** section 2.4) and a small window prompt will appear. Input the threshold values in order of the image sequences in the parent folder, separating numbers by a space. Failure to separate values with a space will result in errors.


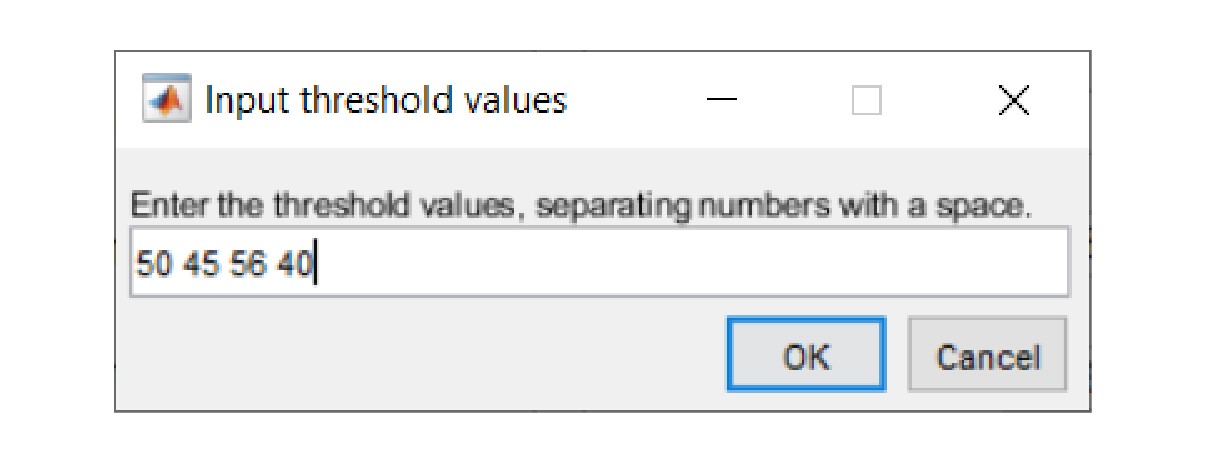


Figure 12: Inputting threshold values for SPA.

This will generate a file in the parent folder called ”lutThresh.m”. Please keep this file as it will be required to run the analysis. If you wish to change any of the threshold values, simply hit the Set button again in ”Set Threshold Values” and it will ask if you wish to overwrite the original values. Or, you can also manually open the file and change any of the parameters and resave.


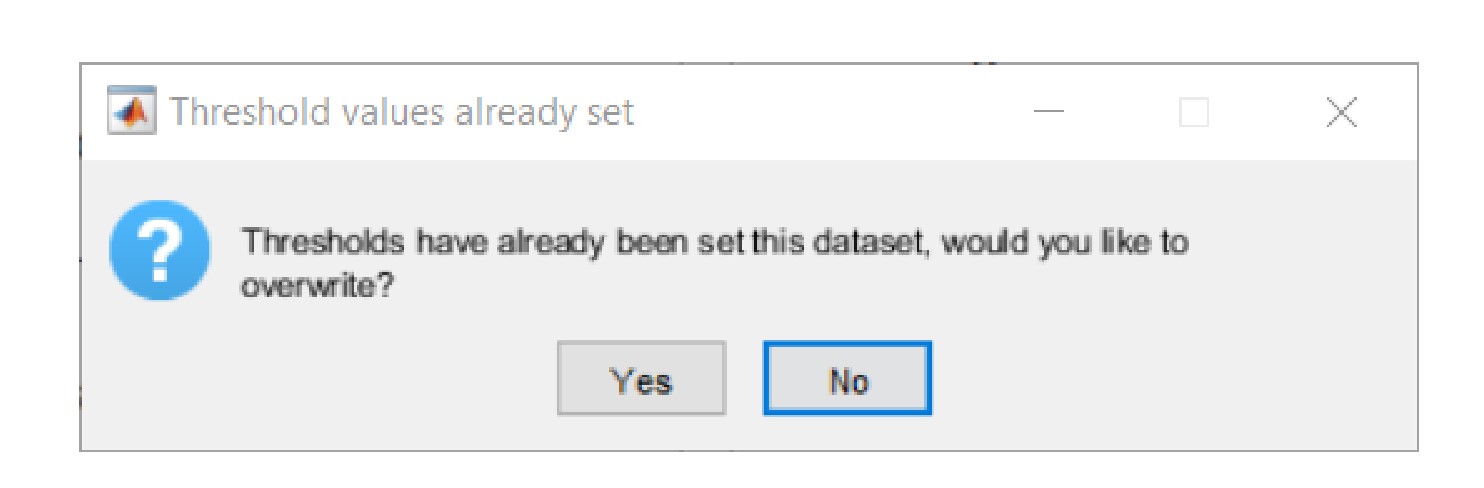


Figure 13: Overwriting and changing original threshold values set in SPA.

## Running analysis and results

After all the parameters have been set, simply hit ”Run” to begin the analysis. Progress bars will appear to let you know how far into the analysis SPA is. Two will be generated for single-day analysis (analysis progress and number of files analysed) and three will be generated for multi-day analysis (analysis progress, number of files analysed, and number of file days analysed).

When the analysis has been set to run, a unique folder with the date and time of analysis will be generated in the parent folder that stores 1) the x,y coordinates of the spots in all frames, 2) the tracking results stored in an .xml format, and 3) the analysis output consisting of the MSD, diffusion coefficients, and the analysis parameters. The unique folder allows for rerunning of analysis several times without overwriting previous results, this is useful for example if you want to see the effects of changing analysis parameters.


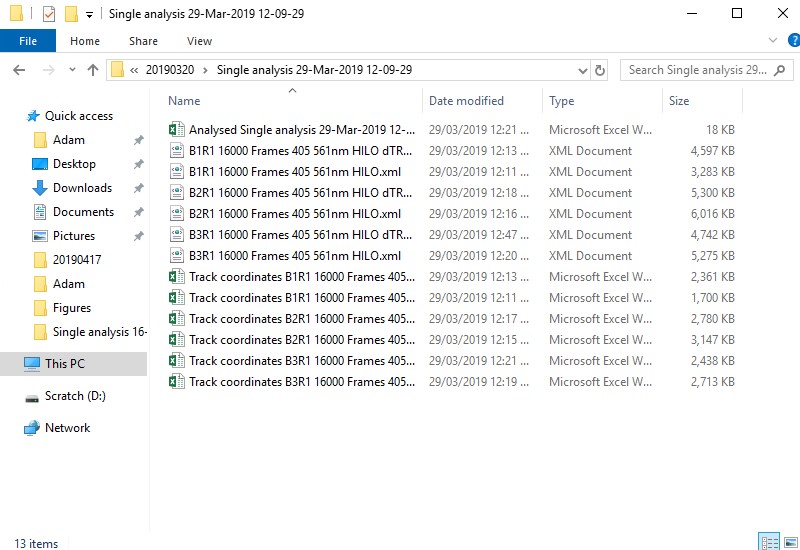


Figure 14: Track coordinates and tracks from SPA analysis.


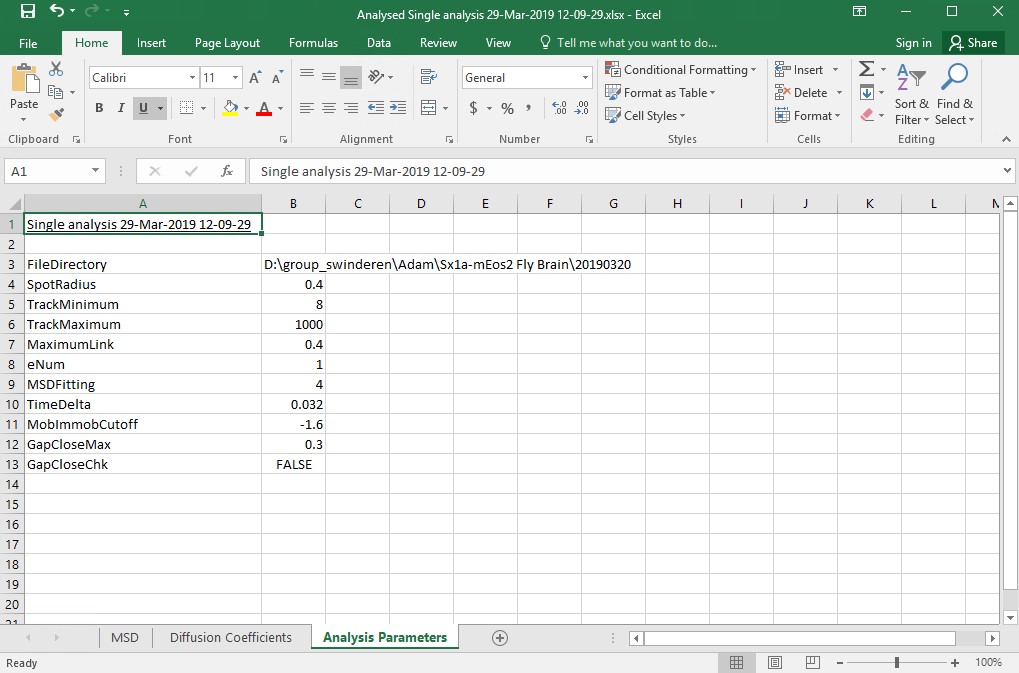


Figure 15: Analysis data from SPA exported to excel spreadsheet.

## Troubleshooting

Here we will detail some common issues that you may run into when trying to run SPA. Please note that this list is not exhaustive and any errors encountered not covered in this section should be reported [here.](https://github.com/AdamDHines/sptPALM-Analysis/issues)

### Java Heap Memory

### Image files too large

# Example Analysis
