# Supplementary figures and images for: Tracking Single Molecule Dynamics in the Adult Drosophila Brain
Source: eNeuro. 2021 May 14;8(3):ENEURO.0057-21.2021. doi: 10.1523/ENEURO.0057-21.2021 (PMC8174007; doi:10.1523/ENEURO.0057-21.2021)

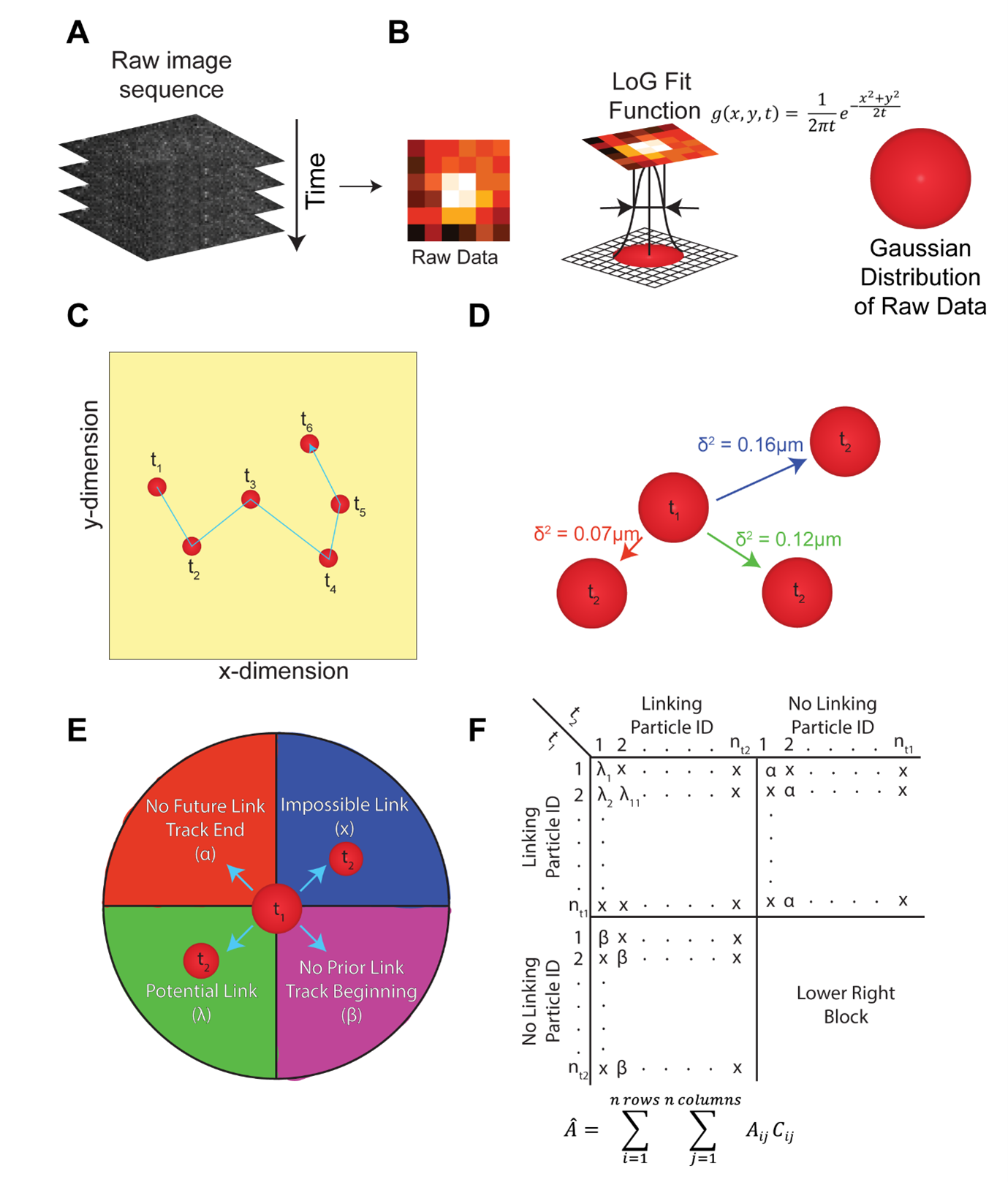

Supplement: Extended Data Figure 1-1 — SPT and PALM. A, B, Raw image sequences from PALM (A) are processed with a LoG convolution filter (B) for automatic spot detection to derive centroids of single Sx1a-mEos2 particles. C, D, Schematic of tracking particles in a 2D sample over time, with links between frames determined based on the relative distance (δ, distance) of a single particle to every other particles (D) from one frame to the next. E, Particle tracking is solved using a LAP cost matrix, where the cost is the relative distance of a particle in frame n to every other particle in frame n + 1. A particle in frame n can have one of four outcomes based on the localization in the proceeding frame. A particle has a potential link (λ) to another particle based on a maximum linking distance which if a particle in the proceeding frame exceeds becomes an impossible link (x). To avoid linking potentially unrelated molecules, it is important to keep stochastic switching of fluorophores light such that molecules detection is sparse. The threshold for the maximum linking distance depends on a variety of factors, including the exposure time of the imaging and the relative speed of the molecule, and if it is membrane bound or cytoplasmic. A particle can also either be the start or the end of a trajectory, and a higher cost value is employed to determine whether a particle should be linked to another particle or not (α and β). F,Example of the cost matrix used to solve SPT. The matrix is solved for least cost to link particles and determine whether a trajectory is at its beginning or its end (adapted from Jaqaman et al., 2008). Download Figure 1-1, TIF file. [file enu-eN-NWR-0057-21-s07.tif]

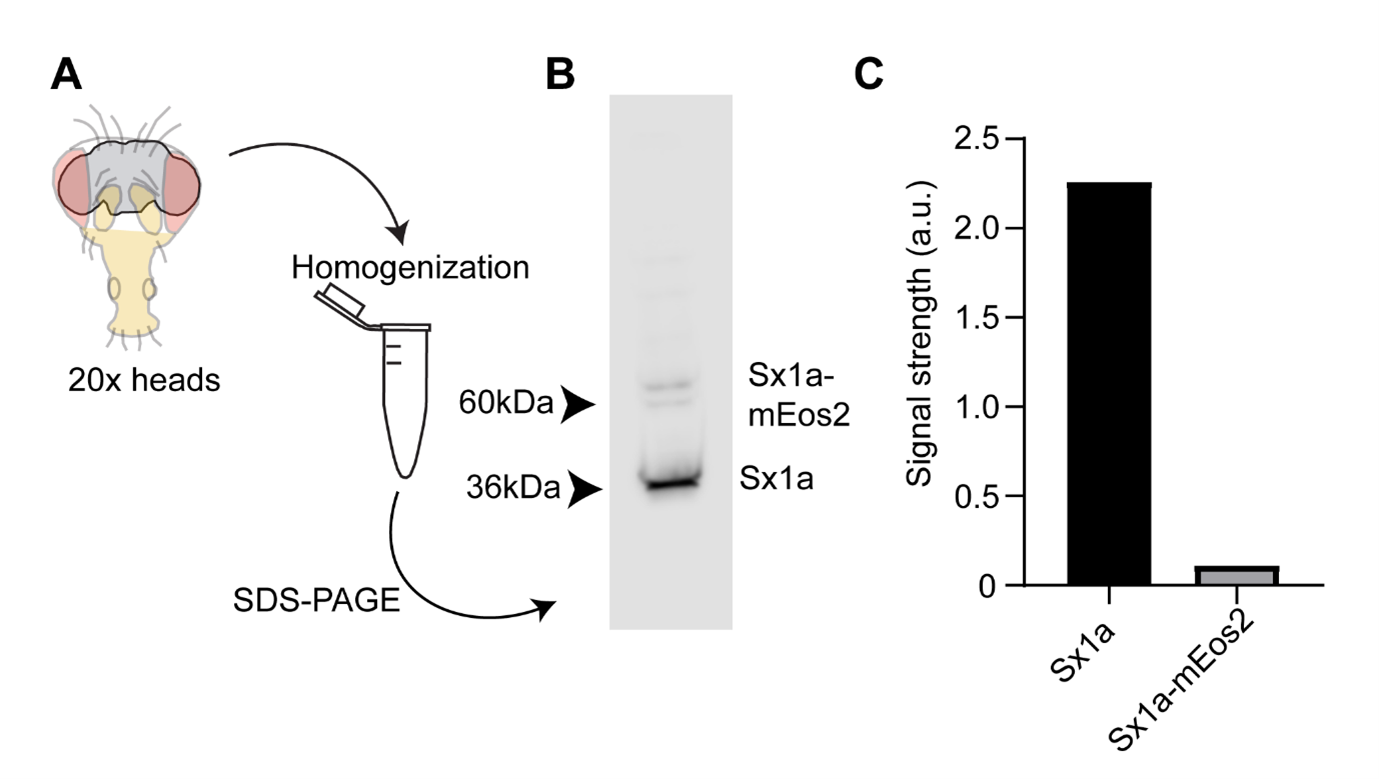

Supplement: Extended Data Figure 1-2 — Quantification of Sx1a-mEos2 expression relative to endogenous Sx1a. A, 20× female heads from experimental flies (w1118;Sx1a-mEos2/+;UAS-TrpA1/R57C10-Gal4) aged 3–5 d were homogenized and run in a Western blot on an SDS-PAGE gel (B) to separate endogenous Sx1a protein (arrow at 36 kDa) from Sx1a-mEos2 (arrow at 60 kDa). C, Quantification of the relative expression of Sx1a-mEos2 compared to endogenous Sx1a shows approximately 8% of the expression level of Sx1a-mEos2. Download Figure 1-2, TIF file. [file enu-eN-NWR-0057-21-s08.tif]

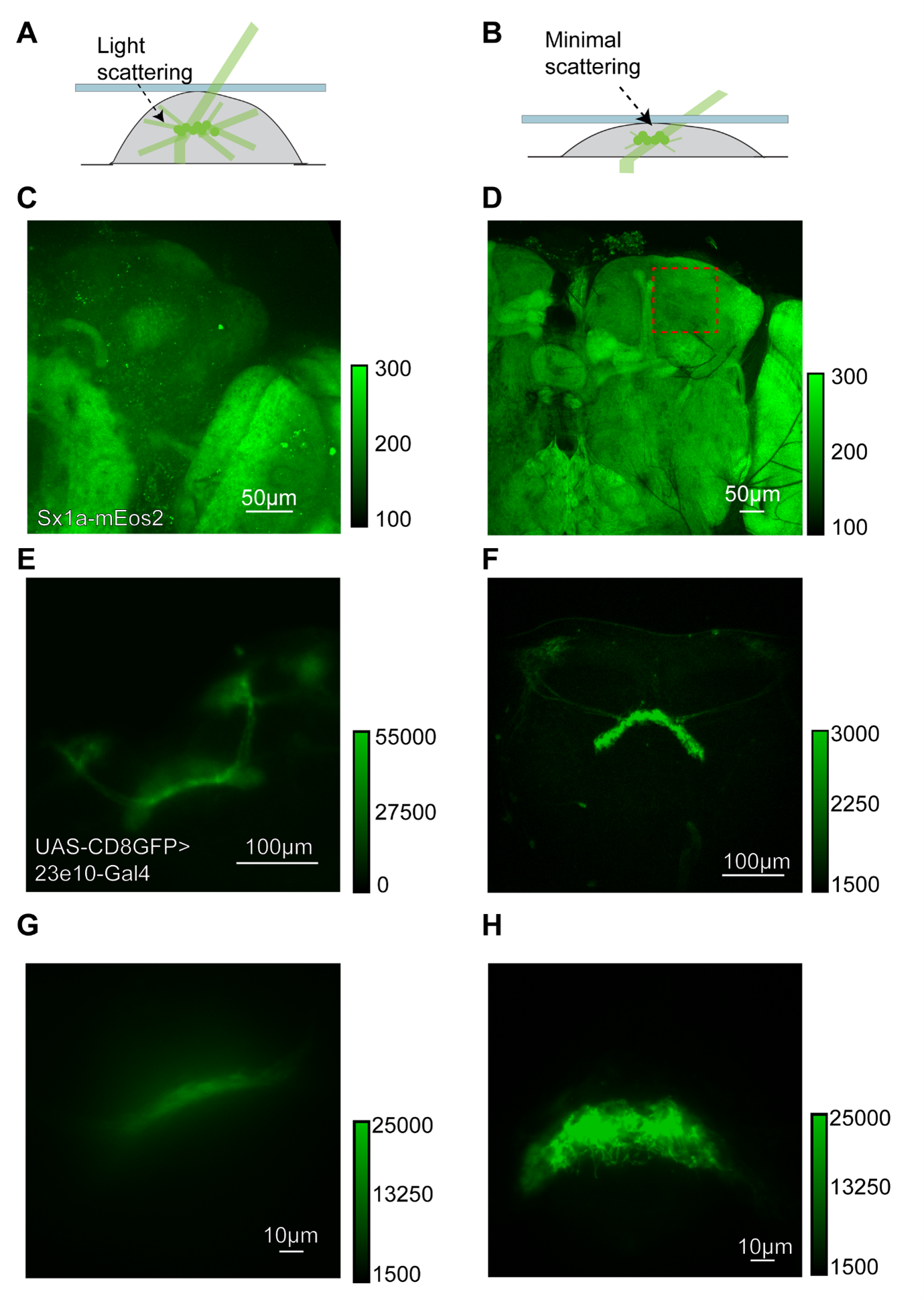

Supplement: Extended Data Figure 1-3 — Internal brain structures remain intact and are better resolved in a compressed preparation. A, When the fly brain is not compressed (thickness = 120 μm), light scattering under a HILO sheet setting decreases the resolution of imaged structures. B, When the brain is lightly compressed (thickness = 40 μm), the scattering interferes less, and structures are more resolved. C, Fly brain expression of Sx1a-mEos2 in an un-compressed preparation shows a distinct lack of neuronal architecture compared to a compressed preparation (D). Red box in D indicates where internally controlled imaging experiments were conducted, in the general vicinity of the lateral protocerebrum.E, G, 10× and 63× oil magnification, respectively, of UAS-CD8GFP>R23E10-Gal4 (Jenett et al., 2012) imaging in a standard uncompressed preparation. F, H, 10× and 63× oil magnification, respectively, of UAS-CD8GFP>R23E10-Gal4 imaging in a compressed preparation, revealing that neural architecture of a defined circuit in the fly brain (the dorsal fan-shaped body) remains intact. Download Figure 1-3, TIF file. [file enu-eN-NWR-0057-21-s09.tif]

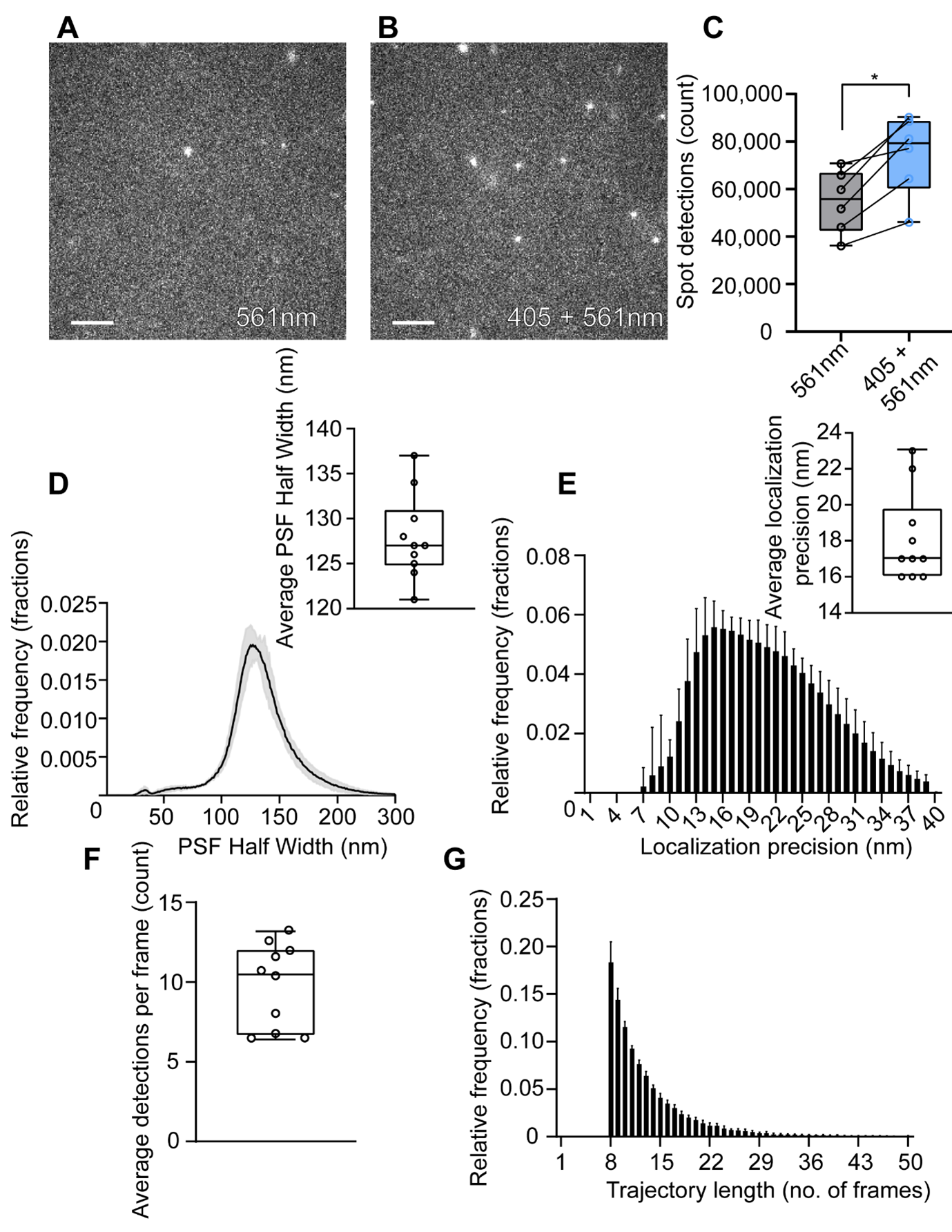

Supplement: Extended Data Figure 1-4 — Analysis of Sx1a-mEos2 localizations in the adult Drosophila brain. A, Single frame from a recording in the fly brain with no 405-nm photoconverting laser while imaging red 561 nm and (B) with the 405-nm photoconversion reveals a (C) significant increase in spot detection (n = 6, average spot detection 561 nm: 54,787 spots, 95% CI 40,901–68,672; average spot detection 405 nm + 561 nm: 74,663 spots, 95% CI 57,049–92,276, p = 0.0313, Wilcoxon test, data is ±5–95th percentile). Spot counts were recorded in the same brain in the same region twice over the course of 8000 frames without the 405-nm laser and another 8000 frames with the 405-nm laser. To analyze the characteristics of Sx1a-mEos2 localizations, recordings in HL3.1 were utilized. Using Zeiss processing software ZEN, we processed the acquired images with PALM, which measured the (D) PSF half width (black line is the average, SD shown in gray) and (E) the localization precision of detected Sx1a-mEos2 molecules. On average, the PSF half width was 127.9 ± a SD of 4.725 nm (n = 10, 95% CI 124.5–131.3 nm), and we achieved on average a localization precision of 18.1 ± a SD of 2.5 nm (n = 10, 95% CI 16.3–19.90 nm). In order to quantify the average number of localizations per frame and trajectory length for Sx1a-mEos2 molecules, we utilized our tracking software SPA (see Materials and Methods). On average, we detected 9.8 ± 2.6 (SD) molecules per frame (n = 10, 95% CI 7.9–11.7) with the majority of trajectory lengths from detected molecules being eight frames long, the minimum required for analysis. All box plots are ±5–95th percentile and histograms are ±SD. Download Figure 1-4, TIF file. [file enu-eN-NWR-0057-21-s10.tif]

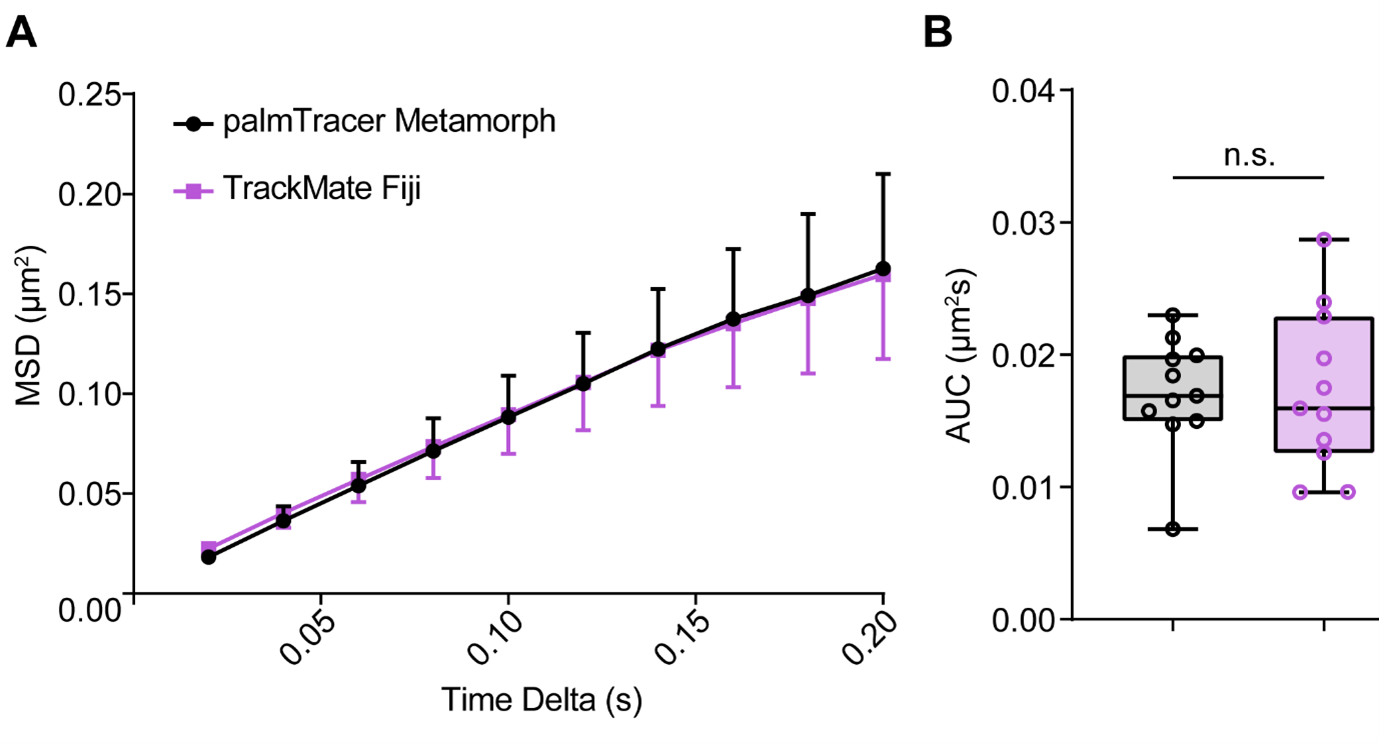

Supplement: Extended Data Figure 1-5 — Validation of the semi-automated SPA script employing TrackMate.To validate the SPA software that was used for all dataanalysis, we employed a known dataset that was analyzed using the Metamorph plugin palmTracer. The data analyzed were derived from rat pheochromocytoma PC12 cells that were transfected with a Munc18-1mEos2 (Kasula et al., 2016)-tagged molecule. sptPALM was performed in the same way, except a lower exposure time of 20 ms was utilized. PC12 cells and Munc18-1mEos2 were provided by Frederic Meunier, Queensland Brain Institute. A, The MSD of Munc18-1mEos2 trajectories and (B) AUC analysis reveals no significant difference between palmTracer and our custom TrackMate analysis scripts (n = 10, p = 0.898, Wilcoxon matched pairs signed-rank test, 95% CI palmTracer 0.0142–0.0200, 95% CI TrackMate 0.0132–0.0213, MSD values presented as ±SD, AUC presented as ±5–95th percentile). Download Figure 1-5, TIF file. [file enu-eN-NWR-0057-21-s11.tif]

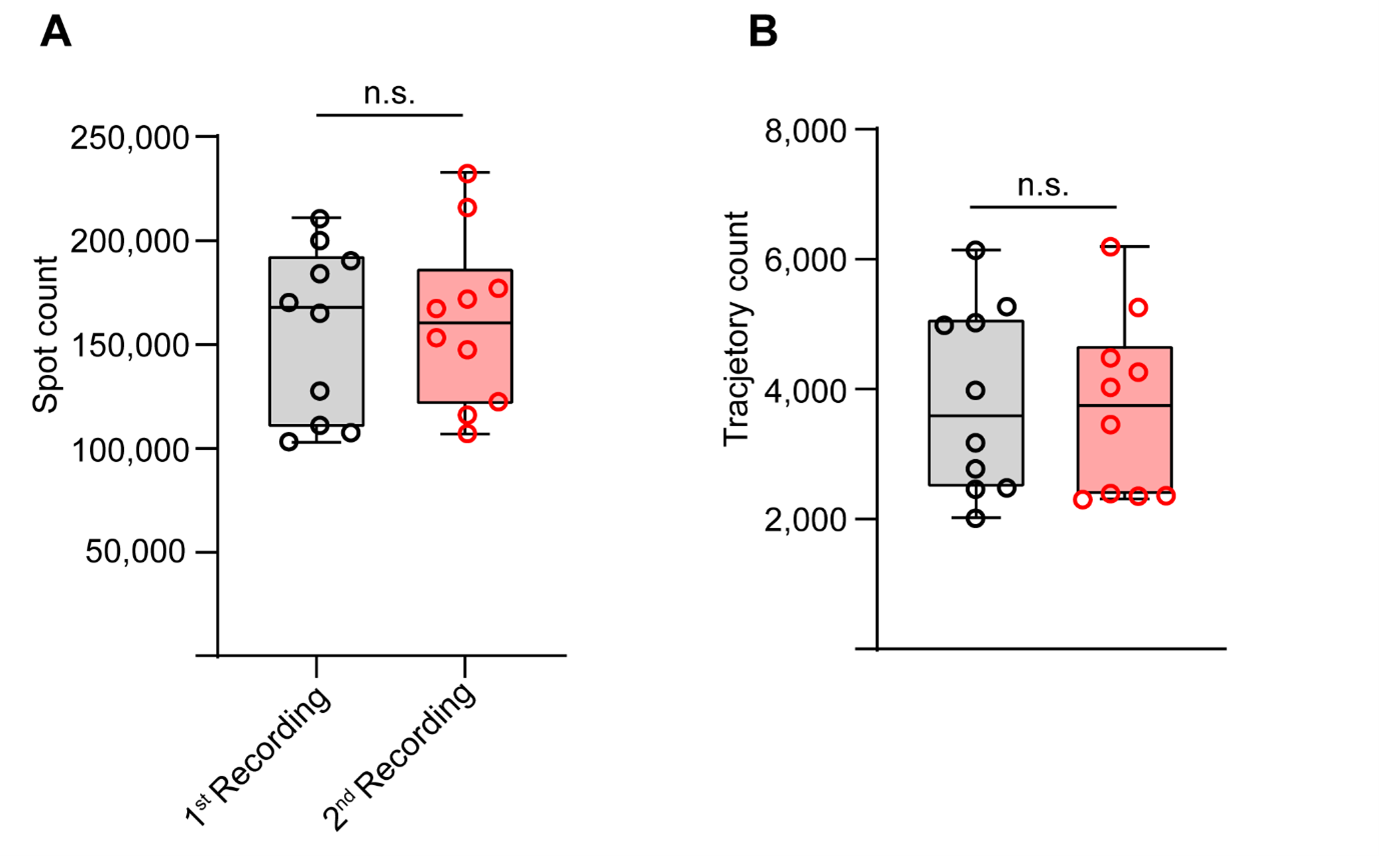

Supplement: Extended Data Figure 2-1 — A, B, Spot and trajectory counts between the first and second recording of Sx1a-mEos2 tracking experiments shows no difference in spot and trajectory counts (data from HL3.1 control recordings, n = 10, spot count p = 0.9118, trajectory count p = 0.7394, n.s., not significant. Statistics performed for both with a Wilcoxon test, data is ±5–95th percentile). Download Figure 2-1, TIF file. [file enu-eN-NWR-0057-21-s12.tif]

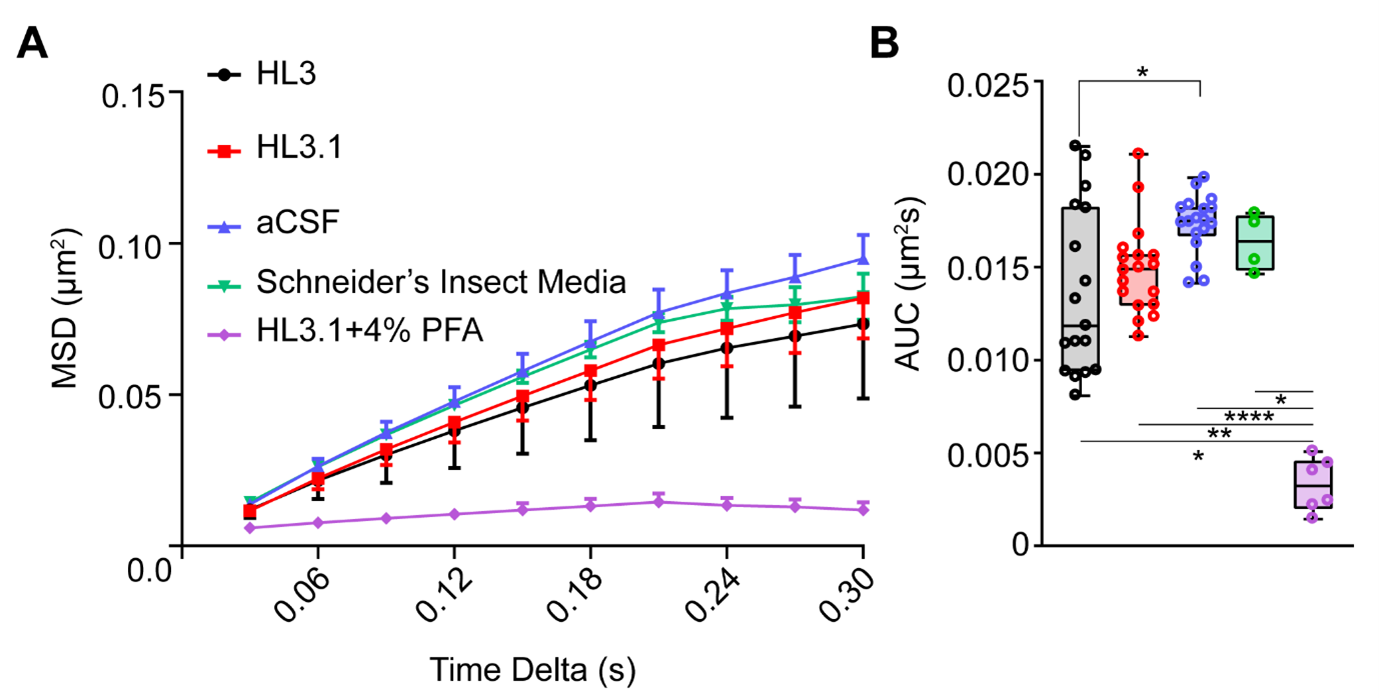

Supplement: Extended Data Figure 3-1 — Comparison of imaging buffers on the mobility of Sx1a-mEos2 particles in the Drosophila brain. During method development phase, several imaging buffers were trialed for physiological relevance and consistency between samples. Three random brain regions were sampled in UAS-dTRPA1>R57C10-Gal4 flies at 30°C for stimulation in either HL3, HL3.1, aCSF, or Schneider’s insect media and compared for their consistency. Also included is a mobility control where brains were fixed in a 4% PFA before imaging in HL3.1 solution, to confirm that tracked molecules are not an artefact of the imaging buffer. A,MSD curves for the average of the three imaging buffers utilized with (B) the AUC highlighting a significant difference between HL3 to aCSF (HL3 n = 6, p = 0.0058, AUC CI 0.0113–0.0160, HL3.1 n = 6, p = 0.0316, AUC CI 0.0137–0.0161, aCSFn = 7, AUC CI 0.0165–0.0181, Schneider’s n = 4, p > 0.999, AUC CI 0.01383–0.01882, Kruskal–Wallis test, MSD data presented as ±SD, AUC data presented as ±5–95th percentile). Despite aCSF providing the best consistency, HL3.1 was selected for its physiological relevance to Drosophila while retaining a degree of consistency above HL3. All imaging buffers were significantly different to the 4% PFA fixed brains, which showed minimal mobility effects (n = 6, p = 0.0212 HL3, p = 0.0067 HL3.1, p < 0.0001 aCSF, p = 0.0116 Schneider’s, AUC CI 0.00174–0.00478, Kruskal–Wallis test). Download Figure 3-1, TIF file. [file enu-eN-NWR-0057-21-s13.tif]

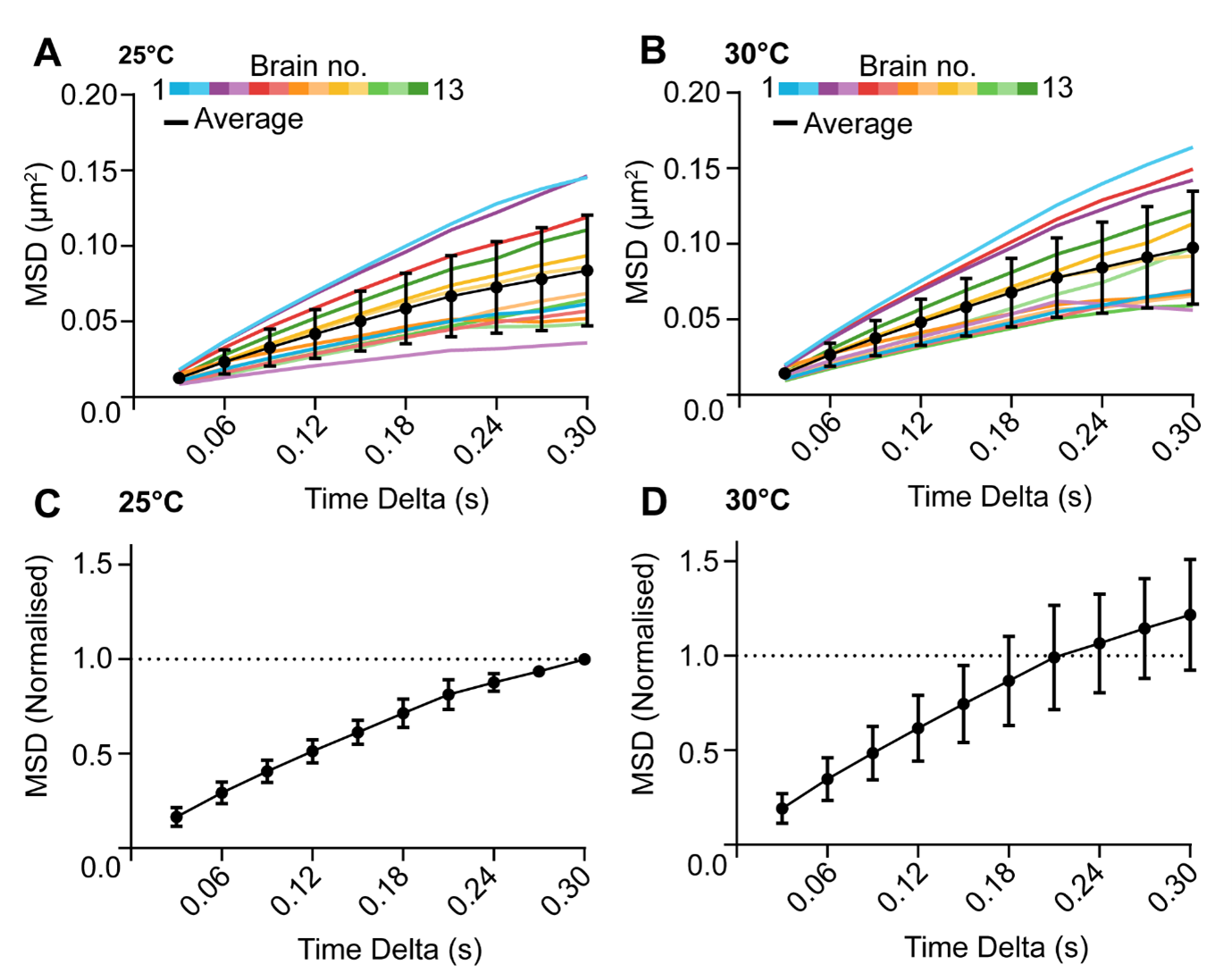

Supplement: Extended Data Figure 3-2 — Normalization of neuronal stimulation MSD curves to baseline. A, Raw and average MSD curves for Sx1a-mEos2 recorded in the adult fly brain at 25°C, each color represents a different brain. B, Raw and average MSD curves in the same brains as A, but at 30°C for an internally controlled paradigm. Note high variance among brains (different colors) but low variance within (same colors). C, Normalized MSD curve for the raw data in A. The peak value of the curve at time point 0.30 (s) was used to normalize each time point, such that the peak of the normalized MSD curve at time 0.30 s is 1.0. D, Normalized MSD curve for the raw data in B, relative to within-brain baseline. Each time point in B was normalized to the matched peak value of the corresponding baseline curves in A. All average data is presented as ±SD. Download Figure 3-2, TIF file. [file enu-eN-NWR-0057-21-s14.tif]

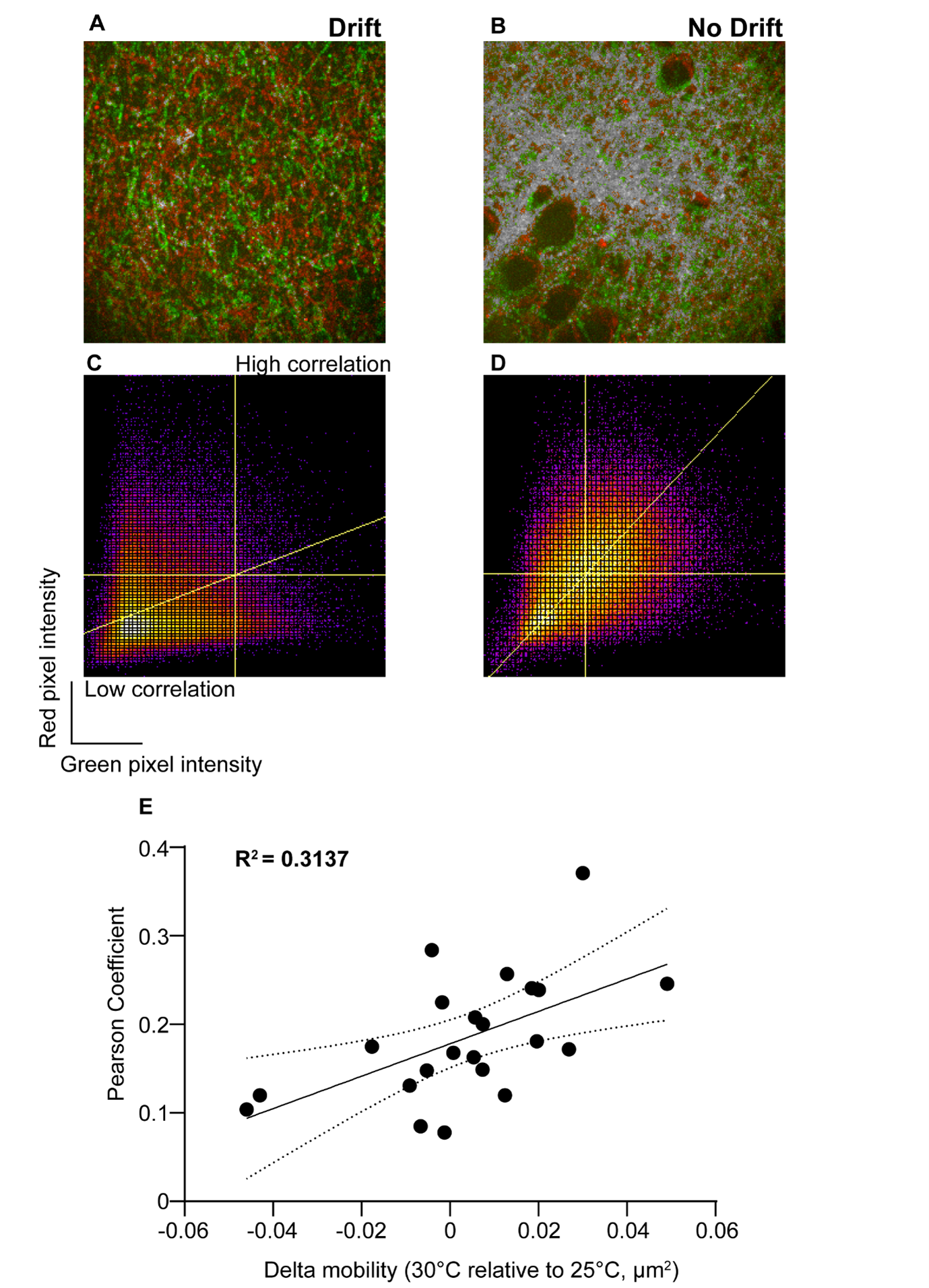

Supplement: Extended Data Figure 3-3 — Correlation between Sx1a-mEos2 mobility and imaging region stability. Dual color images of Sx1a-mEos2 in a single brain comparing the initial 25°C (green) and second 30°C (red) recordings, showing the degree of overlap (gray) in a brain that (A) experienced drift and (B) experienced minimal to no drift. C, D, A Pearson correlation was calculated for 22 brains from the HL3.1 + DMSO condition, showing scatterplots for the degree of pixel correlation between the respective brains in A,B. The pixel intensity of the green and red images in A, B are plotted against one another with the brightness of the correlation scatterplot indicating the degree of overlap between each individual pixel. The four quadrants of the Cartesian plot indicate the distribution of pixel intensity for both the green and red images, with the top right quadrant indicating pixels that are highly correlated and the bottom left quadrant indicating pixels that highly uncorrelated. Regression line is shown. E, Plotting the Pearson coefficients against the change in mobility reveals a correlation between an increase in Sx1a-mEos mobility with TrpA1 stimulation and low drift, whereas brains that drifted have a lower detectable Sx1a-mEos mobility (n = 22, slope = 0.6055, R2 = 0.3137, p = 0.0067, solid line indicates best line of fit, dotted lines indicate 95% CI). Download Figure 3-3, TIF file. [file enu-eN-NWR-0057-21-s15.tif]

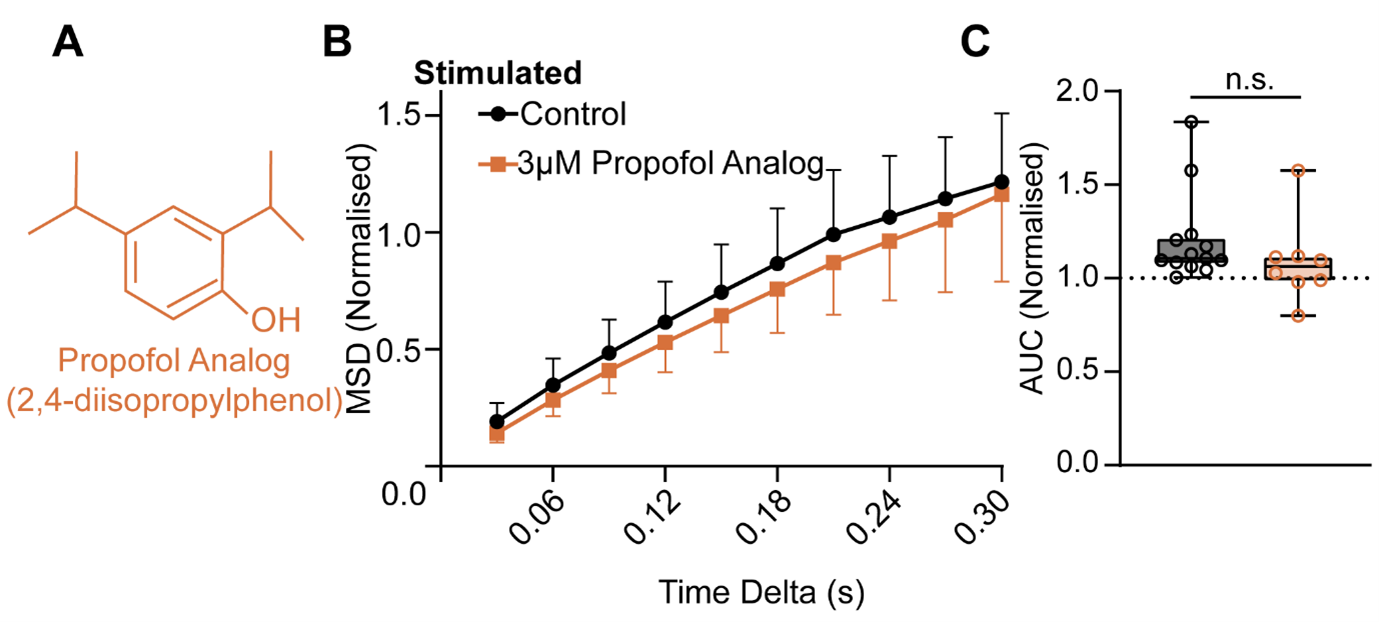

Supplement: Extended Data Figure 4-1 — A structural propofol analog is not able to restrict Sx1a-mEos2 mobility. A, Structure of the non-anesthetic analog of propofol (2,4-diisopropylphenol). Note the change in position of the hydroxyl group on the benzene ring from carbon 1 to carbon 3. B,Under stimulation conditions, the MSD of Sx1a-mEos2 not able to be restricted in the presence of 3 μm of the propofol analog (orange) when compared to DMSO control (black), with no significant change in the (C)AUC (n = 8, p = 0.9866, AUC CI 0.931–1.012, Kruskal–Wallis test, data for MSD is ±SD, data for AUC is ±5–95th percentile). Download Figure 4-1, TIF file. [file enu-eN-NWR-0057-21-s16.tif]

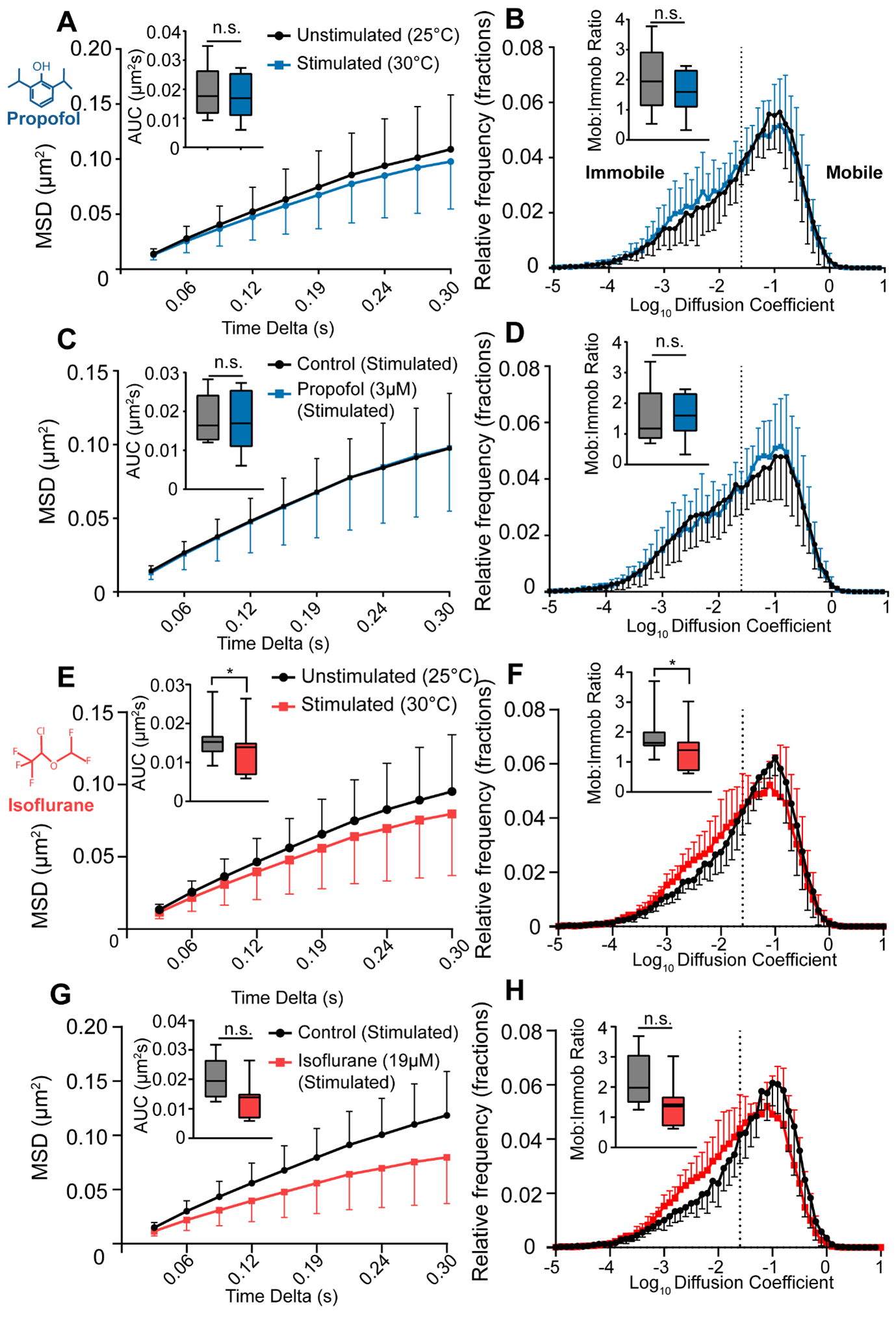

Supplement: Extended Data Figure 4-2 — Non-normalized MSD and diffusion coefficients for Sx1a-mEos2 under propofol and isoflurane. A, Raw MSD and AUC values for Sx1a-mEos under propofol (3 μm) at 25°C (baseline) and 30°C (TrpA1 stimulation) and (B) diffusion coefficients with corresponding mobile-to-immobile ratio (n.s., not significant, Wilcoxon test, paired statistics). C, Raw MSD and AUC values for Sx1a-mEos in TrpA1 stimulated brains (30°C) with and without propofol. D, Diffusion coefficients with corresponding mobile-to-immobile ratio (n.s., not significant, Mann–Whitney test, unpaired statistics). E, Raw MSD and AUC values for Sx1a-mEos under isoflurane (0.19 mm) at 25°C (baseline) and 30°C (TrpA1 stimulation) and (F) diffusion coefficients with corresponding mobile-to-immobile ratio (*p < 0.05, Wilcoxon test, paired statistics). G, Raw MSD and AUC values for Sx1a-mEos in TrpA1 stimulated brains (30°C) with and without isoflurane. H, Diffusion coefficients with corresponding mobile-to-immobile ratio (n.s., not significant, Mann–Whitney test, unpaired statistics). Data are the same as normalized propofol and isoflurane data shown in Figure 4. Download Figure 4-2, TIF file. [file enu-eN-NWR-0057-21-s17.tif]
